# A Theoretically Based Mobile App to Increase Pre-Exposure Prophylaxis Uptake Among Men Who Have Sex With Men: Protocol for a Randomized Controlled Trial

**DOI:** 10.2196/16231

**Published:** 2020-02-21

**Authors:** Jeb Jones, Karen Dominguez, Rob Stephenson, Joanne D Stekler, Amanda D Castel, Leandro A Mena, Samuel M Jenness, Aaron J Siegler, Patrick S Sullivan

**Affiliations:** 1 Department of Epidemiology Rollins School of Public Health Emory University Atlanta, GA United States; 2 Department of Behavioral Sciences and Health Education Rollins School of Public Health Emory University Atlanta, GA United States; 3 Department of Systems, Populations and Leadership School of Nursing University of Michigan Ann Arbor, MI United States; 4 Division of Allergy & Infectious Diseases School of Medicine University of Washington Seattle, WA United States; 5 Department of Epidemiology and Biostatistics Milken Institute School of Public Health The George Washington University Washington, DC United States; 6 Department of Population Health Science John D Bower School of Population Health University of Mississippi Medical Center Jackson, MS United States

**Keywords:** men who have sex with men, pre-exposure prophylaxis, mobile health, electronic health, HIV

## Abstract

**Background:**

HealthMindr is a mobile phone HIV prevention app for men who have sex with men (MSM). In a previous pilot study, HealthMindr was found to be acceptable among users and to demonstrate preliminary effectiveness for increasing pre-exposure prophylaxis (PrEP) uptake among MSM. PrEP is a highly effective HIV prevention intervention; however, uptake remains low.

**Objective:**

The aim of this study will be to assess the efficacy of a mobile app for increasing PrEP uptake among MSM in the southern United States.

**Methods:**

In this randomized controlled trial, we will assess the efficacy of HealthMindr for increasing PrEP uptake among MSM in the following three southern US cities: Atlanta, Georgia; Jackson, Mississippi; and Washington, DC. In total, 657 men will be recruited and randomized to intervention and control arms in a 2:1 ratio. Participants in the intervention arm will receive access to the full HealthMindr app, with information and resources about PrEP (eg, frequently asked questions, risk assessment tool, and PrEP provider locator), other HIV prevention information, ability to order free HIV/sexually transmitted infection test kits, and additional resources related to substance use and mental health. Participants in the control arm will use the HealthMindr app but will only have access to the study timeline and a message center to communicate with study staff. Participants will complete quarterly surveys to assess self-reported PrEP uptake over 12 months of follow-up. Self-reported PrEP uptake will be verified by dried blood spot testing and/or uploading a photograph of a PrEP prescription.

**Results:**

Participant recruitment began in January 2020.

**Conclusions:**

This trial will determine whether the HealthMindr app can increase PrEP uptake among MSM in the southern United States.

**Trial Registration:**

ClinicalTrials.gov NCT03763942; https://clinicaltrials.gov/ct2/show/NCT03763942

**International Registered Report Identifier (IRRID):**

PRR1-10.2196/16231

## Introduction

In the US HIV epidemic, men who have sex with men (MSM) experience disproportionately high HIV prevalence [1-4] and incidence [5-7]. MSM are the only US risk group in which HIV incidence increased after 2000 [6], and the increase is especially alarming among young MSM [5] and MSM of color [8]. MSM aged 13-24 years and 25-34 years are the only MSM groups in which new diagnoses increased after 2009 [9]. The prevalence of HIV infection among MSM is 67 times greater than that among other men in the US population [10]. There are estimated to be more than 4,700,000 MSM in the United States [10], and over 800,000 of these men are estimated to be current candidates for pre-exposure prophylaxis (PrEP) [11].

In the United States, daily oral PrEP is available for MSM as emtricitabine/tenofovir disoproxil fumarate and emtricitabine/tenofovir alafenamide. Despite the effectiveness of PrEP for preventing HIV seroconversion [12,13], there are a number of barriers to PrEP uptake, including cost, awareness, and underestimation of the self-perceived HIV risk [14,15]. Manufacturer payment assistance programs are available to help offset the cost of PrEP; however, these programs do not cover the costs of necessary laboratory tests and can be difficult for patients to navigate [16].

Multiple models of HIV incidence in MSM suggest that to substantially decrease HIV incidence in MSM, it is needed to achieve 40%-50% coverage of multiple prevention services and interventions (eg, condom promotion, HIV testing, PrEP, and treatment as prevention) in at-risk MSM [17-19]. Currently, uptake of many prevention services among MSM is low. HIV testing within the past 12 months, as recommended by some health jurisdictions [20] and the Centers for Disease Control and Prevention (CDC) [21], has been reported by 71% of men in 21 National HIV Behavioral Surveillance cities [22]. However, only 58% of respondents in the American Men’s Internet Survey (AMIS) [23], a national survey that includes more rural MSM and MSM living in smaller cities, reported HIV testing in the past 12 months. In Atlanta, Georgia; Jackson, Mississippi; and Washington, DC, AMIS data indicate that there are significant deficits in annual HIV testing and PrEP knowledge and uptake. Data from the 2017-2018 AMIS surveys indicate a substantial unmet need for PrEP; 51%, 46%, and 58% of HIV-negative MSM in Atlanta, Jackson, and Washington, DC, respectively, are behaviorally eligible for PrEP but have never taken it (unpublished data). Considering the estimated population of HIV-negative MSM in each city [24] and the proportion of PrEP-eligible men and prevalence of PrEP use from AMIS data in each city, we estimate that the unmet need for PrEP among MSM in these three cities is greater than 70,000 MSM.

Electronic health (eHealth) tools provide an opportunity to facilitate uptake of HIV prevention services. A recent summary of eHealth tools for HIV prevention in MSM noted that certain types of prevention services are most amenable to provision through new technologies. Services for which indications can be determined through an algorithm are good candidates to bring to scale with technologies [25]. For example, well-described criteria and algorithms exist to identify individuals with behavioral indications for PrEP [26,27]. Moreover, using technology to administer PrEP eligibility screening could highlight the need for more accessible PrEP services for rural MSM [25]. Furthermore, the indications of men for PrEP change over time [28,29], so efficient use of PrEP among MSM will require periodic reassessment of the HIV risk. Assuming 6-monthly PrEP eligibility screenings for HIV-negative MSM, approximately 8 million PrEP eligibility screenings would be needed in the United States annually to identify PrEP-eligible MSM. Technologies that allow men to conduct periodic self-screening and opt-in to clinical screening when indicated have the potential to substantially reduce health-care system burden.

The HealthMindr app is an eHealth tool designed to promote HIV prevention among young MSM in the United States. HealthMindr is grounded in Social Cognitive Theory [30], particularly the components of self-efficacy, goal setting, outcome expectation, and feedback/self-regulation [31]. HealthMindr is designed to be a comprehensive HIV prevention app with information and links to resources that involve a combination prevention approach (eg, HIV/sexually transmitted infection [STI] testing, PrEP information and provider locators, and condom promotion). Additional details of the app and its components are presented in the Methods. In a pilot study conducted in Atlanta, Georgia and Seattle, Washington, HealthMindr was found to have high acceptability among MSM over a 4-month follow-up [31]. We incorporated feedback from the pilot participants to improve the app, including the inclusion of graphics and videos, additional frequently asked questions (FAQs), and customizable reminders (eg, to order a HIV test and schedule an appointment with a PrEP provider). Although not a primary outcome of the pilot study, 9% of PrEP-eligible participants initiated PrEP over the follow-up period. The primary goal of this study is to assess the efficacy of HealthMindr for increasing PrEP uptake in a randomized controlled trial (RCT) in the southeastern United States. The primary aim of this study is to assess the efficacy of HealthMindr for increasing self-reported PrEP uptake among MSM aged 18-34 years in the three cities of Atlanta, Georgia; Jackson, Mississippi; and Washington, DC in the southern United States, which have a high HIV incidence among young MSM. Participants will be randomized to an intervention arm and provided access to HealthMindr or to a standard-of-care (information only) control arm. Participants in the control arm will be provided access to an app that allows them to manage their study progress and complete surveys but contains no HIV prevention information. The primary outcome will be PrEP uptake, as measured by self-report on quarterly follow-up surveys. PrEP uptake will be verified by dried blood spot testing and/or prescription verification. Secondary outcomes will be to assess app components contributing to PrEP uptake measured via app usage data (ie, paradata), conduct in-depth interviews (IDIs), and measure HIV and STI incidence and PrEP adherence and persistence.

## Methods

### Study Design

We will conduct a mixed-methods RCT with two arms. Participants in the intervention arm will receive access to HealthMindr with enhanced HIV prevention services (HIV test planning and test locator; initial risk/PrEP eligibility assessment; HIV treatment locator; and ordering of free condoms, HIV test kits, and at-home STI specimen collection kits); information about PrEP; PrEP provider locator; initial local PrEP navigator referral; and monthly PrEP eligibility assessment and PrEP navigator referral. The app also provides links to health insurance exchanges, where eligible men can seek health insurance (all cities), Medicaid (DC only), or payment assistance programs. The control arm will be provided standard-of-care HIV prevention and PrEP information upon enrollment and will receive access to the control version of the HealthMindr app. The control version of the app prompts participants to complete a monthly mobile phone–administered attention control survey about diet, exercise, and prescription medications (to assess PrEP uptake) of the same length as that of the monthly PrEP assessment given to the intervention arm in order to maintain the same frequency of contact as that among intervention participants. Participants in the control arm will not have access to the HIV prevention information and services available in HealthMindr used in the intervention arm.

**Figure 1:**
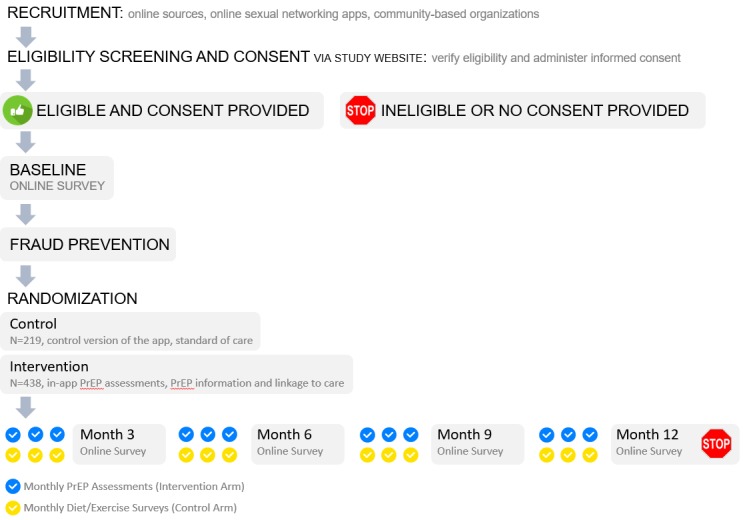
Study schema depicting planned enrollment in each arm and the schedule of surveys and monthly self-assessments. PrEP: pre-exposure prophylaxis.

Study participants will be followed for 12 months, and primary and secondary outcome measurements will be performed at 3-month intervals in both arms. A schematic of the study design is presented in Figure 1. Surveys will be distributed to all participants through the mobile app at baseline and quarterly. Quarterly surveys will take approximately 30 minutes to complete; to limit survey duration, non–time-varying survey elements will not be assessed at each time point. Quarterly surveys will be identical for men in both arms; however, the final survey will include questions on acceptability of HealthMindr only for men in the intervention arm. The surveys are optimized for smartphones to allow them to be completed on participants’ phones. The survey measures represent a comprehensive set of previously validated measures that will characterize the population, allow for assessment of randomization adequacy, document important outcomes, and allow for exploration of possible moderating factors.

During and after follow-up, participants will also be purposively sampled to participate in an IDI to obtain additional insights into the role that HealthMindr plays in decisions to start or not start PrEP during the study period.

### Study App

**Figure 2:**
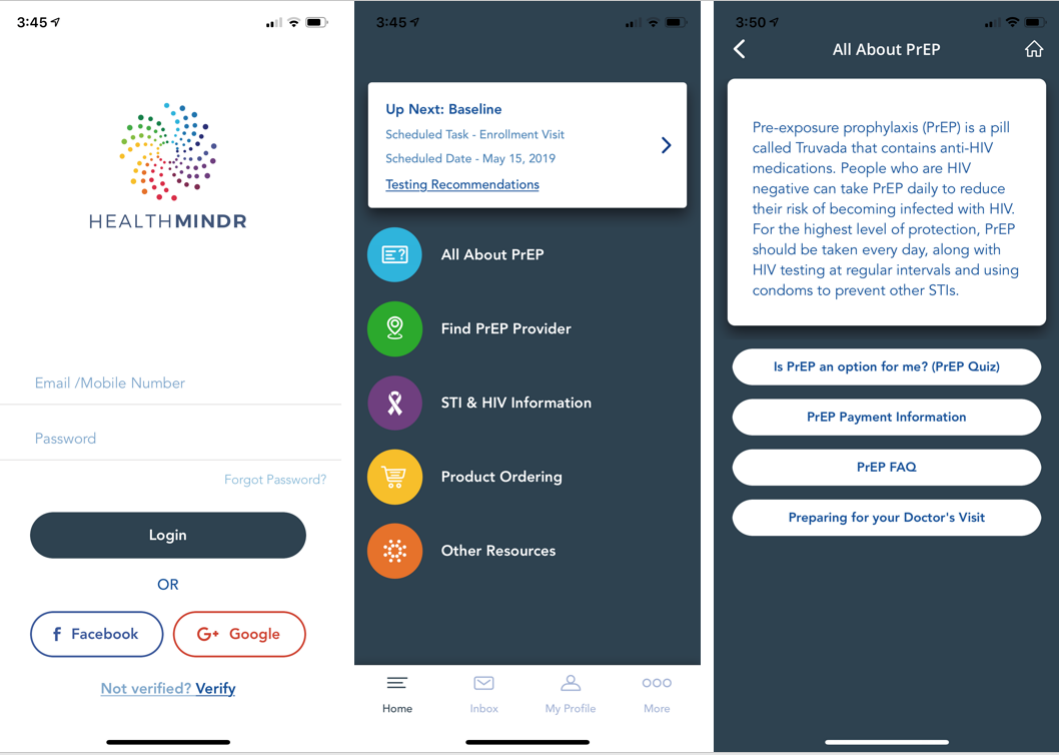
Screenshots of the splash screen, home page, and all about PrEP screen of the HealthMindr app. FAQ: frequently asked questions; PrEP: pre-exposure prophylaxis; STI: sexually transmitted infection.

HealthMindr is a mobile app designed to be a HIV prevention portal for MSM, and it was developed using an iterative community-driven process [32]. As previously described, the app development is grounded in Social Cognitive Theory, particularly the components of self-efficacy, goal setting, outcome expectation, and self-regulation. Participants in the intervention and control arms will download the HealthMindr app (Figure 2). The version of HealthMindr used in this study has been updated and modified according to feedback obtained in a previous pilot study [31]. Following randomization, the HIV prevention features of HealthMindr will become available to participants assigned to the intervention arm. The control version of the app is designed to support an attention control that maintains the same schedule of contact as that among intervention participants and provides a secure method for participants to communicate with study staff. The common features available to all participants and HIV prevention features available to intervention participants are described below.

Participants in the control arm will have access to the control HealthMindr app with limited functionality, which includes the following features:

- Secure messaging: Participants will be able to send and receive secure messages and communicate with study staff. When participants receive a message from study staff, they will have the option of receiving a nonspecific alert (eg, You have a message) on their phone.
- Study timeline: A study timeline will be displayed so that participants can see all study activities (eg, surveys and self-assessments) and seen when they are due.
- Surveys and self-assessments: Participants will be able to follow links to complete all study surveys and self-assessments within the HealthMindr app via a SurveyGizmo API.
- Profile: Participants will be able to create a basic profile to keep their contact information up-to-date.

Participants in the intervention arm will have access to the features described above, as well as the below-mentioned HIV prevention functions. Where appropriate, we have mapped functions to the relevant components of Social Cognitive Theory (eg, goal setting, self-regulation, and self-efficacy).

- HIV testing: Participants will be able to learn more about testing options, plan with built-in reminders to test 2-4 times in the coming year, locate possible places for the tests, and schedule reminders (goal setting).
- Behavioral risk assessment: Participants will be able to complete a short behavioral risk assessment (eg, HIV risk behaviors: sex while drunk or high, unprotected anal intercourse with a positive or unknown HIV status partner, total number of sex partners, recent STI diagnosis, and methamphetamine or popper use and protective behaviors: HIV testing, STI testing, and consistent condom use; feedback/self-regulation). Participants will be prompted to complete the risk assessment monthly but will have the option of completing it at any time on-demand.
- Nonoccupational postexposure prophylaxis (nPEP): Participants will have access to nPEP information, nPEP self-assessment [27], nPEP locator services [33], and PrEP referral for those who evaluate multiple exposures for nPEP indication (goal setting and outcome expectation) [34].
- Product ordering: Participants will be able to order condoms, condom-compatible lubricants, at-home STI specimen collection kits (urethral and rectal gonorrhea and chlamydia, and syphilis; goal setting), and at-home HIV test kits (OraQuick; goal setting). Orders are filled through Amazon fulfillment services.
- Pre-exposure prophylaxis: Participants will have access to PrEP self-assessment at the first app use, monthly rescreening for PrEP eligibility, PrEP recommendations adapted from CDC criteria, PrEP provider locator services, transport or driving directions to providers, and PrEP FAQs. The PrEP self-screener assesses certain key elements of behavioral eligibility (potential indicators of ongoing high risk for HIV acquisition) and recommends potentially eligible MSM to visit PrEP providers for further clinical assessment, with help locating nearby providers and transport directions (self-efficacy, goal setting, and outcome expectations). Locator services will be provided via PrEPLocator.org, which allows users to locate nearby PrEP providers and navigators. PrEP navigation will not be explicitly offered by study staff; however, participants who request PrEP navigation assistance will be directed to resources in their area.
- Substance use and mental health screeners: Participants will have access to brief screeners to conduct self-assessments for anxiety, depression, and substance use problems.
- Substance use and mental health provider directory: Participants will have access to a list of local resources available for treatment, rehabilitation, and counselling. This will allow participants to search the directory of substance use and mental health counseling providers included in the Substance Abuse and Mental Health Services Administration online directory [35].

### Participants and Recruitment

The target enrollment for this study is 657 participants (438 intervention and 219 control participants) across the three study sites. Power analyses are described in detail below. The eligibility criteria for this study are as follows: (1) cisgender male (male at birth and currently identifies as a male); (2) age 18-34 years (inclusive); (3) resident in one of the study metropolitan statistical areas (MSAs; Atlanta, Georgia; Jackson, Mississippi; or Washington, DC); (4) intention to reside in one of the study MSAs for the duration of the trial; (5) available Android or iOS phone with active service and willingness to download the study app; (6) English speaker; (7) report of having anal sex with a man in the past 6 months; and (8) report of being HIV-negative or never having been tested for HIV. Men who report currently being on PrEP will be considered ineligible; however, past PrEP use will not be exclusionary.

Recruitment strategies will aim to recruit MSM who are diverse in terms of race/ethnicity and maintain comparability across sites. Black and Hispanic MSM will be oversampled with the goal of enrolling at least 50% of the study population from these disparately impacted groups. The primary method of recruitment will be via online venues, including social networking sites (eg, Facebook, Instagram, and Twitter), sexual networking apps (eg, Grindr and Scruff), and banner advertisements on websites frequented by MSM (eg, Queerty, Towleroad, and Adam for Adam). In a previous study in Atlanta, Georgia, minimal differences were observed between men recruited online and men recruited via venue-based time-space sampling [36]. We will target men aged at least 18 years, who live in MSAs in Atlanta, Georgia; Jackson, Mississippi; or Washington, DC. Additional metropolitan areas in the southern United States might be added if needed to achieve our recruitment goal. When men click on a banner advertisement, they will be taken to a page containing basic study information that includes a short description of the study activities. If they click on a button to advance, they will progress through screening, consent, and enrollment. These procedures are described below. No user data will be collected from the referral site (eg, Facebook and Grindr); however, unique tracking links will be used to allow identification of the referral site. Recruitment yields from each source will be monitored, and recruitment strategies will be adjusted as needed to reach the target population number.

In addition to online recruitment strategies, participants will be recruited by posting flyers and promoting the study through community partners (eg, community-based organizations and drop-in centers) in the three MSAs. Those recruited through these flyers will be directed to a website for eligibility screening.

### Screening, Consent, and Enrollment

Participants who click on an online recruitment advertisement or follow the URL on a community-based advertisement will be taken to an eligibility screening survey hosted on SurveyGizmo.com, a Health Insurance Portability and Accountability Act-compliant web-based survey platform. Eligible participants will complete an online informed consent form, with the option to view a video summary of the informed consent form [37]. After providing informed consent, participants will be directed to a link to authenticate their smartphone and download the HealthMindr app from either the Google Play Store or Apple App Store. Initially, the app will display a study timeline with a link to the baseline survey, which participants will complete in the app. After the baseline survey is completed, a standardized fraud check procedure will be completed. Participants who pass the fraud check will be randomized and enrolled into the study. Following randomization, the HIV prevention features of HealthMindr will become available to participants assigned to the intervention arm (ie, all participants will have the same HealthMindr app, with additional content made available to participants in the intervention arm).

### Baseline Survey

The baseline survey will take approximately 30 minutes to complete. The measured domains will include demographics; sexual behavior in the past 6 months; PrEP awareness, knowledge, attitude, use, and stigma; HIV/STI testing, diagnosis, and treatment history; health care access and utilization; substance use [38]; intimate partner violence; and mental health. The full survey is included in Multimedia Appendix 1.

### Fraud Prevention Procedures

To avoid fraudulent registrations, the eligibility criteria will not be revealed to prospective participants prior to screening, IP addresses will be assessed to block duplicate screening attempts, and CAPTCHA will be used to prevent bots. Payment of initial baseline incentives will be delayed for 2-3 business days to allow routine checks for duplicate IP addresses, names, email addresses, or phone numbers. Some demographic questions (eg, age and ZIP code) will be repeated in the eligibility screener and baseline survey for verification. All participants will be contacted by phone prior to enrollment. If duplicate contact information or discrepancies between eligibility and baseline survey data are detected, the study staff will attempt to resolve concerns during this phone call and clarify how the situation arose. If duplicate registrations are detected, the records will be inactivated and deemed invalid.

### Randomization

After providing informed consent, downloading the study app, completing the baseline survey, and passing the fraud check, participants will be randomized to the intervention and control arms in a 2:1 ratio (438 intervention and 219 control participants), with stratification by city to ensure balance of the study arms across the three study sites [39]. The 2:1 randomization is designed to ensure that the trial includes enough app users to conduct planned secondary analyses of intervention efficacy according to the level of app usage. Within each city, a computer program will randomly assign each participant to the next treatment allocation from a random-permuted block randomization sequence.

### Follow-Up Survey Schedule

All participants will be asked to complete monthly self-assessments and quarterly surveys. Monthly self-assessments will differ between the study arms. The participants in the intervention arm will complete a PrEP eligibility self-assessment that is intended to promote PrEP uptake (Multimedia Appendix 2). After PrEP initiation, the monthly survey will include the same PrEP eligibility self-assessment to encourage continual awareness of changing HIV risks and four additional questions assessing PrEP adherence. The participants in the control arm will complete an exercise and general health self-assessment (Multimedia Appendix 2). The purpose of the self-assessment in the control arm is to equalize the frequency of participant contact with study procedures across the follow-up period. Quarterly surveys will be used to measure labile demographic characteristics and study outcomes. Participants will receive incentives in the form of electronic gift cards for completion of the baseline and follow-up surveys according to the following schedule: baseline, US $50; months 3 and 6, US $40; month 9, US $50; and month 12, US $60. The graduated increase in the incentive amount over the follow-up period is designed to increase participant retention over the entire follow-up period. Any participant who reports a reactive HIV test result will be contacted to assist with linkage to care.

### Statistical Analysis

The main analysis will use four standard survival analysis techniques to compare the rate of PrEP initiation between the study arms and estimate the effect size and statistical significance for observed differences. This approach will account for loss to follow-up, study termination owing to competing events (eg, HIV seroconversion), and other forms of censoring [40]. The first analysis will use Kaplan-Meier methods to characterize the empirical survival distribution, which is the time-dependent probability of remaining PrEP uninitiated over the follow-up period. This method will yield estimates of the median time to PrEP initiation and cumulative incidence with 95% confidence intervals. In the second analysis, log-rank or related (eg, Harrington-Fleming) statistical tests will be used to test the null hypothesis of no difference in the rate of PrEP initiation between the study arms, with a cutoff *P* value of .05 used to determine significance [41]. In the third analysis, a Cox proportional hazards model will be used to estimate the hazard ratio associated with the study arms in order to characterize the strength of the causal effect of app use. The primary model will include an independent exposure variable for the study arms as randomized (ie, standard intent-to-treat analysis), and covariates of interest (including those related to the four domains along the PrEP continuum) will be included in the model to increase precision [42]. In the fourth analysis, parametric survival models (eg, under exponential or Weibull distributional assumptions) will be used to characterize the rate of PrEP initiation overall and by study arm. Participants will be analyzed according to the randomized arm (ie, intention-to-treat analysis).

### Primary Outcome

The primary outcome will be the rate of PrEP uptake as measured by self-report in the app-based surveys at months 3, 6, 9, and 12. Additionally, all participants reporting PrEP initiation will be asked to submit confirmation of self-report either by laboratory testing for the presence of tenofovir diphosphate via dried blood spots submitted by mail or uploading a photograph of their PrEP prescription or pill bottle via HealthMindr. Tenofovir diphosphate, although conventionally used as a measure of PrEP adherence, will be interpreted as a qualitative (yes/no) indicator of having taken PrEP. Participants who opt to verify their PrEP uptake will be incentivized with an electronic gift card for submitting a dried blood spot (US $40 incentive) or uploading a photograph of their prescription or pill bottle (US $15 incentive).

### Secondary Outcomes

App paradata (eg, number of unique times the app is accessed and time spent on each app page) will be recorded on the server side when users access app content. To prevent overestimation of the time spent in the app and to protect participant privacy, the app will automatically log out after 3 minutes of inactivity whether left in the foreground or background of the mobile phone. The server will record a log of all activities the user completes in the app. These data will be used to summarize typical usage patterns of the app, including frequency of overall app usage; frequency of use of individual app components, including monthly check-in surveys to assess PrEP eligibility; and typical time spent on the app. These data will also be used to inform secondary per-protocol analyses of intervention efficacy.

HIV and STI diagnoses will be assessed in multiple ways. Participants in the intervention arm will be able to order HIV and STI at-home test kits. Those who order HIV test kits will be asked to self-report their results via the app. STI test kits will contain biospecimen collection tools that participants will use to self-collect samples to test for syphilis and pharyngeal, rectal, and urethral gonorrhea and chlamydia. STI test kits will contain prepaid envelopes that participants will use to return samples for laboratory testing. STI results will be provided to participants by study staff. Negative results will be delivered via in-app messages, and positive results will be delivered via a phone call. Staff will attempt to link participants having positive STI diagnoses to treatment. Participants in the intervention arm will be able to report HIV/STI tests received outside of the context of the study. Finally, all participants will be asked about recent HIV/STI diagnoses at the baseline and quarterly surveys.

Among participants initiating PrEP, we will assess self-reported adherence (ie, number of doses missed in the past 30 days) and persistence (ie, time on PrEP) at each quarterly survey.

### Power Analyses

Power calculations are conducted for the main analysis of time to PrEP initiation comparing the intervention arm with a control condition. Regarding estimates of PrEP uptake in four quarters spanning 2014 to 2015, 4507 men started PrEP in the United States [43]. Assuming all of these men are MSM, which is an overestimate, and using the number of MSM estimated by the CDC to be eligible for PrEP as the denominator [11], only 0.6% (4507/813,970) of eligible MSM started PrEP between mid-2014 and mid-2015. Thus, our assumption that at most 2% of men in the control arm will initiate PrEP during the study period is conservative. According to our preliminary pilot data, men using the app initiated PrEP at a rate of 9% in a 4-month period, and we conservatively estimate that men in the intervention period would initiate PrEP at a rate of 9% in a year. We will enroll 438 intervention and 219 control participants, which will provide 91% power (α=.05) to detect a 4.5-fold increase in the rate of uptake in the intervention arm when compared with the control arm. If the effects were less than our conservative estimates, we would retain reasonable power (4-fold: 80% power; 3.5-fold: 74% power). These power calculations assume 20% annual attrition.

The power calculations mentioned above were conducted at the time the study was proposed. However, recent data from the AMIS [44] indicate that approximately 3% of MSM initiated PrEP in the past year in the three study MSAs (data unpublished). Changing the calculations above to reflect 3% uptake among control participants, we would have 69% power to detect an intervention effect.

### In-Depth Interviews

Up to 30 IDIs (approximately 10 per site) will be conducted within 1 month of PrEP initiation among participants in the intervention arm who start PrEP. Up to 20 additional IDIs will be conducted within 1 month of study completion among participants who fit the following criteria (maximum of five for each criterion): initiated PrEP, did not initiate PrEP, initiated PrEP but stopped PrEP prior to the end of follow-up, and cycled on and off PrEP during follow-up. Participants undergoing IDIs will be purposively sampled to ensure representation of men who access the app at least once during follow-up and receive a PrEP recommendation. Participants who initiate PrEP and are interviewed after 1 month of follow-up will not be eligible to be reinterviewed at the end of the study. The IDIs will be conducted by trained qualitative interviewers and will follow semistructured interview guides. The IDIs will assess the extent to which app components facilitated or inhibited PrEP uptake. Central to the IDIs will be understanding the participants’ use of the app. Participants who initiate PrEP will be asked to describe the role that the app played in their decision and which app components were most integral to their decision. Participants who do not initiate PrEP will be asked if there are additional components (eg, better linkage to providers and other information) that could be included in the app, which might cause them to change their mind about initiating PrEP. HealthMindr is designed to address known barriers, including individual risk perception (via monthly PrEP self-assessments) and financial costs (via PrEP FAQs and payment information), and participants will be asked to comment on whether they accessed these sections of the app. Participants who do not initiate PrEP will be asked to demonstrate how they used the app and which components of the app were not used. Participants who stop and do not restart PrEP or who cycle on and off PrEP during follow-up will be asked to describe how HealthMindr influenced their decision to initiate PrEP and whether additional functions or support might have influenced their decision to discontinue PrEP.

Additional IDIs will be attempted with participants who report testing HIV-positive during follow-up. These IDIs will follow a structure similar to that described above. Additional questions will be added to explore the factors related to PrEP and seroconversion. Interviews will explore attitudes toward HIV prevention generally and PrEP specifically. Participants will be asked to comment on why they did or did not use PrEP and mention any barriers to PrEP they might have experienced.

### Qualitative Analysis

All IDIs will be digitally recorded, transcribed verbatim, and deidentified. Transcripts will be entered into MAXQDA (VERBI GmbH, Berlin, Germany), which facilitates the processes of coding, annotating, and retrieving text such that analysts might note patterns in the textual data across themes. Data analyses will be conducted using a phenomenological inquiry framework [45,46]. Phenomenology is focused on describing what a given group of people have in common when they experience a phenomenon and is an inductive analytic approach that allows the patterns, themes, and categories of analysis to emerge from data [45,46]. Data are then presented through textual phenomena descriptions based on summaries of experiences described by respondents. Composite descriptions offer explanations of underlying structures that exist across the respondents’ experiences [45,47].

### Trial Registration and Institutional Review Board Approval

This study has been reviewed and approved by the Institutional Review Board at Emory University (#IRB00102006), and the Institutional Review Boards at George Washington University and University of Mississippi Medical Center have agreed to rely on this decision. This study has been registered at ClinicalTrials.gov (NCT03763942).

## Results

Participant enrollment began in January 2020. Study results are expected to be available in 2022.

## Discussion

PrEP is a highly effective HIV prevention intervention, and with widespread use and high levels of adherence, it has the potential to drastically reduce HIV incidence among MSM in the United States. Despite its promise, PrEP uptake has been slow since receiving Food and Drug Administration approval in 2012 [48,49]. Its uptake has been the slowest among population groups that are most disproportionately affected by the HIV epidemic, including black and Hispanic MSM [50,51]. Innovative interventions that can be brought to scale are needed to increase PrEP uptake among disproportionately impacted populations.

HealthMindr is innovative in its approach of promoting PrEP in the context of a package of HIV prevention information and services delivered via a theory-based app. In addition to PrEP-specific content, HealthMindr provides information on HIV/STI testing, nPEP, and condom use; allows ordering of HIV/STI test kits, condoms, and personal lubricants; provides substance use and mental health resources; and provides service locators for STI/HIV testing and substance use and mental health counselors. These components form the backbone of a combination HIV prevention approach and provide multiple motivations for users to return to the app over time.

This study is subject to limitations and challenges. Despite our intention to oversample MSM of color, including Latinx MSM, HealthMindr is only available in English. Thus, language barriers might prevent otherwise eligible men from participating in this study. Currently, only oral tenofovir disoproxil fumarate/emtricitabine and emtricitabine plus tenofovir alafenamide are approved for use as PrEP in the United States. Approval of other formulations or modalities (eg, injectables) may necessitate changes to the protocol for the measurement and verification of PrEP uptake.

The results of this study will be useful for understanding the extent to which a mobile app designed to promote HIV prevention interventions for MSM, with a focus on PrEP, can increase PrEP uptake. If effective, as our preliminary data indicate, this smartphone-based intervention will be scalable to increase PrEP access to MSM across the United States.

## Appendix

Multimedia Appendix 1 Baseline survey for the HealthMindr randomized controlled trial.

Multimedia Appendix 2 Monthly check-in surveys to assess pre-exposure prophylaxis eligibility (intervention arm) and diet and exercise (control arm).

## Abbreviations

AMIS: American Men’s Internet Survey
CDC: Centers for Disease Control and Prevention
FAQ: frequently asked question
IDI: in-depth interview
MSA: metropolitan statistical area
MSM: men who have sex with men
nPEP: nonoccupational postexposure prophylaxis
PrEP: pre-exposure prophylaxis
RCT: randomized controlled trial
STI: sexually transmitted infection
