# Supplementary material for: A Theoretically Based Mobile App to Increase Pre-Exposure Prophylaxis Uptake Among Men Who Have Sex With Men: Protocol for a Randomized Controlled Trial
Source: JMIR Res Protoc. 2020 Feb 21;9(2):e16231. doi: 10.2196/16231 (PMC7060494; doi:10.2196/16231)
Supplement: Multimedia Appendix 1 [file resprot_v9i2e16231_app1.docx]

# HealthMindr Pilot Baseline Survey

## Intro

### Welcome to the HealthMindr study! Please complete the following survey. It will take you about 30 minutes to complete.  We are going to be asking questions about various topics, including your background, life experience, healthcare, your sexual partners and relationships, and substance use. Questions marked with a red asterisk (*) are required questions that you must answer to move forward. This is a forward-only survey. When you finish a page, proceed to the next page by clicking the "Next" button. You may not go backwards to pages you already have completed. If you feel like you have made a mistake, please contact the study team. Your privacy is important to us. All information you provide in this survey will be held confidentially. Your answers will be used only for research purposes.

###

## Demographics: Age

Validation: %s format expected

Shortname / Alias: dob

### What is your date of birth? (enter MM/DD/YYYY)*

_________________________________________________

Validation: Must be numeric Whole numbers only Positive numbers only

**Logic: Hidden by default**

Shortname / Alias: age

### Age:

_________________________________________________

**Page entry logic:** This page will show when: Question "Age:" is less than "18"

## Demographics - Confirm Age if <18

**Logic: Hidden by default**

### ****We are unable to move you through the survey. Please contact the HealthMindr study team for assistance.****

Validation: %s format expected

Shortname / Alias: confdob

### Please confirm your date of birth (enter MM/DD/YYYY).*

_________________________________________________

Validation: Must be numeric Whole numbers only Positive numbers only

**Logic: Hidden by defaultShow/hide trigger exists.**

Shortname / Alias: confage

### Confirm Age:

_________________________________________________

## Email Action for <18 Respondent

## Demographics: Race & Ethnicity

**Logic: Show/hide trigger exists.**

Shortname / Alias: hispanic

#### Do you consider yourself Hispanic or Latino?

( ) Yes

( ) No

**Logic: Hidden unless: #3 Question "Do you consider yourself Hispanic or Latino?" is one of the following answers ("Yes")**

Shortname / Alias: ancestry

#### What best describes your Hispanic or Latino ancestry? (Check all that apply)

[ ] Cuban

[ ] Dominican

[ ] Mexican

[ ] Puerto Rican

[ ] Other, specify:: _________________________________________________

Shortname / Alias: race

#### What is your race? (Check all that apply)*

[ ] American Indian or Alaska Native

[ ] Asian

[ ] Black or African American

[ ] Native Hawaiian or Other Pacific Islander

[ ] White

[ ] Other, specify:: _________________________________________________

## Demographics: Area of Residence

Validation: %s format expected Using custom RegEx pattern

Shortname / Alias: zipcode

### What is the 5-digit ZIP code for the location where you primarily live?*

_________________________________________________

## Demographics: Education

**Logic: Show/hide trigger exists.**

Shortname / Alias: currenroll

#### Are you enrolled in school? "School" could mean a school or program where you are working toward a high school diploma, GED, or college/technical degree.

( ) Yes

( ) No

**Logic: Hidden unless: #7 Question "Are you enrolled in school?
"School" could mean a school or program where you are working toward a high school diploma, GED, or college/technical degree." is one of the following answers ("Yes")**

Shortname / Alias: curreducat

#### What is your current grade level in school?

( ) High school

( ) GED program

( ) Technical or vocational school

( ) Two-year college

( ) Four-year college

( ) Graduate school

**Logic: Hidden unless: #7 Question "Are you enrolled in school?
"School" could mean a school or program where you are working toward a high school diploma, GED, or college/technical degree." is one of the following answers ("No")**

Shortname / Alias: hleducat

#### What is the highest level of school that you have completed?

( ) None, no formal schooling

( ) 6th-8th grade

( ) 9th-11th grade

( ) High school diploma

( ) High school certificate of completion (no diploma)

( ) GED

( ) Some college, technical school, or vocational school

( ) Technical or vocational school graduate

( ) Two-year college graduate

( ) Four-year college graduate

( ) Some graduate school

( ) Master's degree or above

## Demographics: Current Employment, Health Insurance Status

**Logic: Show/hide trigger exists.**

Shortname / Alias: employed

#### Are you currently employed?

( ) Yes

( ) No

**Logic: Hidden unless: #10 Question "Are you currently employed?" is one of the following answers ("Yes")**

Shortname / Alias: empl_ftpt

#### Are you employed full-time or part-time? Part-time means that you work less than 35 hours per week during most weeks.

( ) Full-time

( ) Part-time

## Demographics: Marital Status

**Logic: Show/hide trigger exists.**

Shortname / Alias: rltn_male

#### Are you currently in a committed relationship with a male partner? (Someone that you feel committed to above all others--this is someone you might call your boyfriend, significant other, or life partner.)

( ) Yes

( ) No

( ) Don't know

**Logic: Show/hide trigger exists. Hidden unless: #12 Question "Are you currently in a committed relationship with a male partner?
(Someone that you feel committed to above all others--this is someone you might call your boyfriend, significant other, or life partner.)" is one of the following answers ("Yes","Don't know")**

Shortname / Alias: marital

#### Which of the following best describes your current marital status?

( ) Legally married

( ) Registered domestic partnership or civil union

( ) Widowed

( ) Divorced

( ) Separated

( ) Never married

**Logic: Hidden unless: #13 Question "Which of the following best describes your current marital status?" is one of the following answers ("Legally married","Registered domestic partnership or civil union","Widowed","Divorced","Separated")**

Shortname / Alias: marital_partner

#### Is/Was this partner:

( ) Male

( ) Female

( ) Transgender female or transfeminine

( ) Transgender male or transmasculine

## Demographics: Income, Sexual Identity

Shortname / Alias: income

#### What was your household income last year from all sources before taxes? (monthly/yearly)

( ) $0 to $417 (monthly) / $0 to $4,999 (yearly)

( ) $418 to $833 (monthly) / $5,000 to $9,999 (yearly)

( ) $834 to $1,250 (monthly) / $10,000 to $14,999 (yearly)

( ) $1,251 to $1,667 (monthly) / $15,000 to $19,999 (yearly)

( ) $1,668 to $2,500 (monthly) / $20,000 to $29,999 (yearly)

( ) $2,501 to $3,333 (monthly) / $30,000 to $39,999 (yearly)

( ) $3,334 to $4,167 (monthly) / $40,000 to $49,999 (yearly)

( ) $4,168 to $6,250 (monthly) / $50,000 to $74,999 (yearly)

( ) $6,251 or more (monthly) / $75,000 or more (yearly)

( ) Don't know

Validation: Min = 1 Must be numeric Whole numbers only Positive numbers only

Shortname / Alias: depend

### Including yourself, how many people did this income support?

_________________________________________________

Shortname / Alias: sexid

#### Do you think of yourself as:

( ) Heterosexual or straight

( ) Homosexual or gay

( ) Bisexual

( ) Other, please specify:: _________________________________________________

## Demographics: Housing Stability

Shortname / Alias: currlive

#### Where do you currently live?

( ) Your own house or apartment

( ) Temporarily in a home with friends or relatives

( ) Hotel

( ) A car

( ) A shelter

( ) Group home

( ) Supportive services housing

( ) On the street

( ) With a parent or guardian

( ) Other

**Logic: Show/hide trigger exists.**

Shortname / Alias: housing

#### Which of these best describes your current housing situation?

( ) Stable/permanent

( ) Transitional/temporary

( ) Homeless

( ) Other, specify:: _________________________________________________

( ) Don't know

**Logic: Hidden unless: #19 Question "Which of these best describes your current housing situation?" is one of the following answers ("Homeless")**

Shortname / Alias: homeless_livedw

#### Who were you living with most recently before you became homeless?

( ) Family member(s)

( ) Friend(s)/Roommate(s)

( ) Partner/Spouse/Significant Other

( ) Lived alone

**Logic: Hidden unless: #19 Question "Which of these best describes your current housing situation?" is one of the following answers ("Homeless")**

Shortname / Alias: homeless_reason

#### What do you think is the main reason you are currently homeless?

( ) Employment/financial issues

( ) Family not supportive of me coming out as gay or bisexual

( ) Break-up/other issue with significant other

( ) Drug/alcohol issues

( ) Issues related to living with HIV

( ) Other, please specify:: _________________________________________________

## Demographics: Housing Stability (cont'd)

Shortname / Alias: housing_worry

#### How much do you worry about your current or future housing situation?

( ) Not at all

( ) A little

( ) A lot

**Logic: Show/hide trigger exists.**

Shortname / Alias: movedp6m

#### Have you moved in the past 6 months? (By "moved" we mean you changed your residence.)

( ) Yes

( ) No

**Logic: Hidden unless: #23 Question "Have you moved in the past 6 months?
(By "moved" we mean you changed your residence.)" is one of the following answers ("Yes")**

Shortname / Alias: timesmovedp6m

#### How many times have you moved in the past 6 months?

( ) 1

( ) 2

( ) 3

( ) More than 3 times

## Demographics: Food Insecurity

**Logic: Show/hide trigger exists.**

Shortname / Alias: foodsec_p12m

#### In the past 12 months, did you or other adults in the household ever cut the size of your meals or skip meals because there wasn't enough money for food?

( ) Yes

( ) No

( ) Prefer not to answer

( ) Don't know

**Logic: Hidden unless: #25 Question "In the past 12 months, did you or other adults in the household ever cut the size of your meals or skip meals because there wasn't enough money for food?" is one of the following answers ("Yes")**

Shortname / Alias: foodsec_freq

#### How often did this happen?

( ) Almost every month

( ) Some months but not every month

( ) Only 1 or 2 months

( ) Prefer not to answer

( ) Don't know

## Demographics: Incarceration

**Logic: Show/hide trigger exists.**

Shortname / Alias: everarrest

#### Have you ever been arrested?

( ) Yes

( ) No

**Logic: Show/hide trigger exists. Hidden unless: #27 Question "Have you ever been arrested?" is one of the following answers ("Yes")**

Shortname / Alias: jail_p12m

#### In the past 12 months, have you spent one or more nights in jail or prison? (Check all that apply)

[ ] Jail

[ ] Prison

[ ] Neither of these

**Logic: Hidden by default**

### *****You entered a future date for the month/year you most recently spent one or more nights in jail or prison. Please correct this.*****

**Logic: Hidden by default**

### *****The date you most recently spent one or more nights in jail or prison cannot be before your birth date ([question('value'), id='3']). Please correct this.*****

Validation: Min = 1 Must be numeric Whole numbers only Positive numbers only

**Logic: Hidden unless: #28 Question "In the past 12 months, have you spent one or more nights in jail or prison? (Check all that apply)" is one of the following answers ("Jail","Prison")**

Shortname / Alias: timesjail_p12m

### In the past 12 months, on how many separate occasions did you spend one or more nights in jail or prison?

_________________________________________________

**Logic: Hidden unless: #28 Question "In the past 12 months, have you spent one or more nights in jail or prison? (Check all that apply)" is one of the following answers ("Jail","Prison")**

Shortname / Alias: yr_jail

#### What was the most recent month and year you spent one or more nights in jail or prison? (If you don't know the exact month, please enter your best guess.) Year:

( ) 1992

( ) 1993

( ) 1994

( ) 1995

( ) 1996

( ) 1997

( ) 1998

( ) 1999

( ) 2000

( ) 2001

( ) 2002

( ) 2003

( ) 2004

( ) 2005

( ) 2006

( ) 2007

( ) 2008

( ) 2009

( ) 2010

( ) 2011

( ) 2012

( ) 2013

( ) 2014

( ) 2015

( ) 2016

( ) 2017

( ) 2018

( ) 2019

( ) 2020

( ) 2021

**Logic: Hidden unless: #28 Question "In the past 12 months, have you spent one or more nights in jail or prison? (Check all that apply)" is one of the following answers ("Jail","Prison")**

Shortname / Alias: mo_jail

#### Month:

( ) January

( ) February

( ) March

( ) April

( ) May

( ) June

( ) July

( ) August

( ) September

( ) October

( ) November

( ) December

## Transition btw Demographics & Sex Hx

### This next section will ask some questions about your sexual behavior and partners. Please be aware of the time period we are asking before you answer the question.

### Let's get started!

###

###

## Sexual Behavior: Partner History, Past 6 Mos

**Page exit logic:** No partners in the past 6 months, skip to HIV-negative cascade**IF:** #32 Question "**During the last 6 months**, did you have sex with a person who was:" is one of the following answers ("I did not have sex in the past 6 months") **THEN:** Jump to [page 35 - Transition btw Sex Hx & HIV-negative Cascade](#Page98)

**Logic: Show/hide trigger exists.**

Shortname / Alias: partsex_p6m

#### ****During the last 6 months****, did you have sex with a person who was:

[ ] Male

[ ] Female

[ ] Transgender female or transfeminine

[ ] Transgender male or transmasculine

[ ] I did not have sex in the past 6 months

Validation: Min = 1 Must be numeric Whole numbers only Positive numbers only

**Logic: Hidden unless: #32 Question "During the last 6 months, did you have sex with a person who was:" is one of the following answers ("Male")**

Shortname / Alias: malepart_p6m

### ****During the last 6 months****, with how many different men did you have anal or oral sex?

_________________________________________________

**Page entry logic:** This page will show when: #33 Question "**During the last 6 months**, with how many different men did you have anal or oral sex?" is greater than "0"

## Sexual Behavior: Partner History, Past 6 Mos (cont'd)

**Logic: Hidden by default**

### *****The number of partners you had anal sex with is greater than the total number of partners over the past 6 months. Please correct this.*****

Validation: Must be numeric Whole numbers only Positive numbers only

Shortname / Alias: numaip6m

### Of the [question('value'), id='43'] male partner(s) you had sex with ****in the last 6 months****, how many did you have anal sex with?

_________________________________________________

Shortname / Alias: male_exchange_p6m

#### Were any of these male sex partners an exchange partner - this is, a partner that you have sex with in exchange for money, drugs, food, or something else of value?

( ) Yes

( ) No

( ) Don't know

## Number of Oral Sex Partners, P6M (hidden)

## Sexual Behavior: Partner History: Past 6 Mos (cont'd)

Validation: Min = 1 Must be numeric Whole numbers only Positive numbers only

**Logic: Show/hide trigger exists. Hidden unless: #32 Question "During the last 6 months, did you have sex with a person who was:" is one of the following answers ("Female")**

Shortname / Alias: fempart_p6m

### ****During the last 6 months****, with how many women did you have vaginal or anal sex?

_________________________________________________

**Logic: Hidden unless: #36 Question "During the last 6 months, with how many women did you have vaginal or anal sex?" is greater than "0"**

Shortname / Alias: fem_exchange_p6m

#### Were any of these female sex partners an exchange partner - that is, a partner that you have sex with in exchange for money, drugs, food, or something else of value?

( ) Yes

( ) No

( ) Don't know

Validation: Min = 1 Must be numeric Whole numbers only Positive numbers only

**Logic: Show/hide trigger exists. Hidden unless: #32 Question "During the last 6 months, did you have sex with a person who was:" is one of the following answers ("Transgender female or transfeminine")**

Shortname / Alias: twpart_p6m

### ****During the last 6 months****, with how many transfeminine individuals did you have sex?

_________________________________________________

**Logic: Hidden unless: #38 Question "During the last 6 months, with how many transfeminine individuals did you have sex?" is greater than "0"**

Shortname / Alias: tw_exchange_p6m

#### Were any of these transfeminine individuals an exchange partner - that is, a partner that you have sex with in exchange for money, drugs, food, or something else of value?

( ) Yes

( ) No

( ) Don't know

Validation: Min = 1 Must be numeric Whole numbers only Positive numbers only

**Logic: Show/hide trigger exists. Hidden unless: #32 Question "During the last 6 months, did you have sex with a person who was:" is one of the following answers ("Transgender male or transmasculine")**

Shortname / Alias: tmpart_p6m

### ****During the last 6 months****, with how many transmasculine individuals did you have sex?

_________________________________________________

**Logic: Hidden unless: #40 Question "During the last 6 months, with how many transmasculine individuals did you have sex?" is greater than "0"**

Shortname / Alias: tm_exchange_p6m

#### Were any of these transmasculine individuals an exchange partner - that is, a partner that you have sex with in exchange for money, drugs, food, or something else of value?

( ) Yes

( ) No

( ) Don't know

**Logic: Hidden unless: (((#35 Question "Were any of these male sex partners an exchange partner - this is, a partner that you have sex with in exchange for money, drugs, food, or something else of value?" is one of the following answers ("Yes") OR #37 Question "Were any of these female sex partners an exchange partner - that is, a partner that you have sex with in exchange for money, drugs, food, or something else of value?" is one of the following answers ("Yes")) OR #39 Question "Were any of these transfeminine individuals an exchange partner - that is, a partner that you have sex with in exchange for money, drugs, food, or something else of value?" is one of the following answers ("Yes")) OR #41 Question "Were any of these transmasculine individuals an exchange partner - that is, a partner that you have sex with in exchange for money, drugs, food, or something else of value?" is one of the following answers ("Yes"))**

Shortname / Alias: exchange_detail

#### You said you exchanged sex for money, drugs, food, or something else of value. Do you receive goods/money, give goods/money or both?

( ) I received goods and/or money

( ) I gave goods and/or money

( ) Both

**Page entry logic:** This page will show when: #33 Question "**During the last 6 months**, with how many different men did you have anal or oral sex?" is exactly equal to "1"

## Sexual Behavior: 1 male partner, p6m

Shortname / Alias: mainmpart

#### Earlier you told us you have had one male partner in the past 6 months. Is/was this partner a main partner? A main partner is someone that you feel committed to above all others -- this is someone you might call your boyfriend, significant other, or life partner.

( ) Yes

( ) No

Shortname / Alias: rltn_m_active

#### Next, think of whether you are currently in an active, ongoing sexual relationship with your male partner. By that, we mean that you generally have oral or anal sex at least once per month and you expect to continue doing so for some time. Is the relationship with this partner active and ongoing?

( ) Yes

( ) No

( ) Don't know

**Logic: Show/hide trigger exists. Hidden unless: (#43 Question "Earlier you told us you have had one male partner in the past 6 months. Is/was this partner a main partner? A main partner is someone that you feel committed to above all others -- this is someone you might call your boyfriend, significant other, or life partner." is one of the following answers ("No") AND #44 Question "Next, think of whether you are currently in an active, ongoing sexual relationship with your male partner. By that, we mean that you generally have oral or anal sex at least once per month and you expect to continue doing so for some time. Is the relationship with this partner active and ongoing?" is one of the following answers ("No"))**

Shortname / Alias: rltn_m_once

#### Was this a one-time partner?

( ) Yes

( ) No

**Logic: Hidden unless: #45 Question "Was this a one-time partner?" is one of the following answers ("No")**

Shortname / Alias: aip6m_once

#### In the last 6 months, how often did you have anal sex with this partner?

( ) Less than once per month

( ) Once per month

( ) Once per week

( ) More than once per week

**Page entry logic:** This page will show when: #33 Question "**During the last 6 months**, with how many different men did you have anal or oral sex?" is exactly equal to "1"

## Sexual Behavior: 1 Male Partner, p6m (cont'd)

**Logic: Show/hide trigger exists.**

Shortname / Alias: onepart_aitype

#### In the last 6 months, which of the following did you do with this partner? (Check all that apply.)

[ ] Receptive anal sex (you bottomed with him)

[ ] Insertive anal sex (you topped with him)

**Logic: Hidden unless: #47 Question "In the last 6 months, which of the following did you do with this partner?
(Check all that apply.)" is one of the following answers ("Receptive anal sex (you bottomed with him)")**

Shortname / Alias: onepart_bcondom

#### When you had receptive (you bottomed) anal sex with this partner over the past 6 months, how often were you fully protected by a condom? This means you or your partner used a condom the entire time you had sex, and the condom did not break or fall off.

( ) Never

( ) Rarely

( ) Sometimes

( ) Often

( ) Always

**Logic: Hidden unless: #47 Question "In the last 6 months, which of the following did you do with this partner?
(Check all that apply.)" is one of the following answers ("Insertive anal sex (you topped with him)")**

Shortname / Alias: onepart_tcondom

#### When you had insertive (you topped) anal sex with this partner over the past 6 months, how often were you fully protected by a condom? This means you or your partner used a condom the entire time you had sex, and the condom did not break or fall off.

( ) Never

( ) Rarely

( ) Sometimes

( ) Often

( ) Always

**Page entry logic:** This page will show when: #33 Question "**During the last 6 months**, with how many different men did you have anal or oral sex?" is greater than "1"

## Sexual Behavior: Multiple Male Partners, p6m

Validation: Must be numeric Whole numbers only Positive numbers only

Shortname / Alias: mult_m_active

### Earlier you told us you have had [question('value'), id='43'] male partners in the past 6 months. How many of your partners are ****active and ongoing****? By that, we mean that you generally have oral or anal sex at least once per month and you expect to continue doing so for some time.

_________________________________________________

**Logic: Show/hide trigger exists.**

Shortname / Alias: mult_m_mainpart

#### Are/were any of these [question('value'), id='43'] partners a ****main partner****? A main partner is someone that you feel committed to above all others - this is someone you might call your boyfriend, significant other, or spouse.

( ) Yes

( ) No

Validation: Min = 1 Must be numeric Whole numbers only Positive numbers only

**Logic: Hidden unless: #51 Question "Are/were any of these [question('value'), id='43'] partners a main partner? A main partner is someone that you feel committed to above all others - this is someone you might call your boyfriend, significant other, or spouse." is one of the following answers ("Yes")**

Shortname / Alias: num_mainpart

###

_________________________________________________

## Calculating remaining sex partners, p6m

**Page entry logic:** This page will show when: (#33 Question "**During the last 6 months**, with how many different men did you have anal or oral sex?" is greater than "1" AND remainp6m is greater than "0")

## Sexual Behavior: Multiple Male Partners, p6m (cont'd)

**Logic: Hidden by default**

### ****The total number of partners you reported does not equal the number of male partners you reported earlier ([question('value'), id='43']).****

Validation: Must be numeric Whole numbers only Positive numbers only

Shortname / Alias: num_once

### You've told us that you had [question("value"), id="43"] total male partners in the past 6 months, of whom [question("value"), id="66"] were main partners. For the remaining [question("value"), id="69"] partners who were not main, how many did you have sex with only one time?

_________________________________________________

## Checking Partner Totals

**Page entry logic:** This page will show when: (#43 Question "Earlier you told us you have had one male partner in the past 6 months. Is/was this partner a main partner? A main partner is someone that you feel committed to above all others -- this is someone you might call your boyfriend, significant other, or life partner." is one of the following answers ("Yes") OR #51 Question "Are/were any of these [question('value'), id='43'] partners a **main partner**? A main partner is someone that you feel committed to above all others - this is someone you might call your boyfriend, significant other, or spouse." is one of the following answers ("Yes"))

## Sexual Behavior: At least 1 Main Partner

### For the next few questions we are interested in hearing about your main partner(s) over the past 6 months.

Shortname / Alias: multpart_aifreq

#### ****In the last 6 months****, how often did you have anal sex with your main partner(s)?

( ) Less than once per month

( ) Once per month

( ) Once per week

( ) More than once per week

**Logic: Show/hide trigger exists.**

Shortname / Alias: multpart_aitype

#### ****In the last 6 months****, which of the following did you do with your main partner(s)? (Check all that apply.)

[ ] Receptive anal sex (you bottomed with him)

[ ] Insertive anal sex (you topped with him)

**Logic: Hidden unless: #55 Question "In the last 6 months, which of the following did you do with your main partner(s)? (Check all that apply.)" is one of the following answers ("Receptive anal sex (you bottomed with him)")**

Shortname / Alias: multpart_bcondom

#### When you had receptive (you bottomed) anal sex with this partner over the past 6 months, how often were you fully protected by a condom? This means you or your partner used a condom the entire time you had sex, and the condom did not break or fall off.

( ) Never

( ) Rarely

( ) Sometimes

( ) Often

( ) Always

**Logic: Hidden unless: #55 Question "In the last 6 months, which of the following did you do with your main partner(s)? (Check all that apply.)" is one of the following answers ("Insertive anal sex (you topped with him)")**

Shortname / Alias: multpart_tcondom

#### When you had insertive (you topped) anal sex with this partner over the past 6 months, how often were you fully protected by a condom? This means you or your partner used a condom the entire time you had sex, and the condom did not break or fall off.

( ) Never

( ) Rarely

( ) Sometimes

( ) Often

( ) Always

**Page entry logic:** This page will show when: (#43 Question "Earlier you told us you have had one male partner in the past 6 months. Is/was this partner a main partner? A main partner is someone that you feel committed to above all others -- this is someone you might call your boyfriend, significant other, or life partner." is one of the following answers ("No") AND remainp6m is greater than or equal to "1")

## Sexual Behavior: At least 1 Casual Partner

### For the next few questions we are interested in hearing about your non-main partners over the past 6 months.

Shortname / Alias: caspart_aifreq

#### ****In the last 6 months****, how often did you have anal sex with your non-main partner(s)?

( ) Less than once per month

( ) Once per month

( ) Once per week

( ) More than once per week

**Logic: Show/hide trigger exists.**

Shortname / Alias: caspart_aitype

#### ****In the last 6 months****, which of the following did you do with your non-main partner(s)? (Check all that apply)

[ ] Receptive anal sex (you bottomed with him)

[ ] Insertive anal sex (you topped with him)

**Logic: Hidden unless: #59 Question "In the last 6 months, which of the following did you do with your non-main partner(s)? (Check all that apply)" is one of the following answers ("Receptive anal sex (you bottomed with him)","Versatile (you bottomed and topped with him)")**

Shortname / Alias: caspart_bcondom

#### When you had receptive (you bottomed) anal sex with this partner over the past 6 months, how often were you fully protected by a condom? This means you or your partner used a condom the entire time you had sex, and the condom did not break or fall off.

( ) Never

( ) Rarely

( ) Sometimes

( ) Often

( ) Always

**Logic: Hidden unless: #59 Question "In the last 6 months, which of the following did you do with your non-main partner(s)? (Check all that apply)" is one of the following answers ("Insertive anal sex (you topped with him)","Versatile (you bottomed and topped with him)")**

Shortname / Alias: caspart_tcondom

#### When you had insertive (you topped) anal sex with this partner over the past 6 months, how often were you fully protected by a condom? This means you or your partner used a condom the entire time you had sex, and the condom did not break or fall off.

( ) Never

( ) Rarely

( ) Sometimes

( ) Often

( ) Always

**Page entry logic:** This page will show when: #33 Question "**During the last 6 months**, with how many different men did you have anal or oral sex?" is greater than or equal to "1"

## Sexual Behavior: HIV-Positive Partners

**Logic: Show/hide trigger exists.**

Shortname / Alias: hivpart_p6m

#### ****In the past 6 months****, have you had sex with any partners who you knew to be HIV-positive?

( ) Yes

( ) No

( ) Don't know

**Logic: Hidden by default**

### *****The number of HIV-positive partners entered is greater than the total number of partners over the past 6 months. Please correct this.*****

Validation: Min = 1 Must be numeric Whole numbers only Positive numbers only

**Logic: Hidden unless: #62 Question "In the past 6 months, have you had sex with any partners who you knew to be HIV-positive?" is one of the following answers ("Yes")**

Shortname / Alias: numpospart

### ****In the past 6 months****, how many of your partners were HIV-positive?

_________________________________________________

Validation: Must be numeric Whole numbers only Positive numbers only

**Logic: Hidden unless: #62 Question "In the past 6 months, have you had sex with any partners who you knew to be HIV-positive?" is one of the following answers ("Yes")**

Shortname / Alias: posparttop

### ****In the past 6 months****, how many times did you have insertive anal sex (you were the top) with an HIV-positive partner?

_________________________________________________

Validation: Must be numeric Whole numbers only Positive numbers only

**Logic: Hidden unless: #62 Question "In the past 6 months, have you had sex with any partners who you knew to be HIV-positive?" is one of the following answers ("Yes")**

Shortname / Alias: pospartbottom

### ****In the past 6 months****, how many times did you have receptive anal sex (you were the bottom) with an HIV-positive partner?

_________________________________________________

**Logic: Hidden unless: #62 Question "In the past 6 months, have you had sex with any partners who you knew to be HIV-positive?" is one of the following answers ("Yes")**

Shortname / Alias: undetectable

#### ****In the past 6 months****, did any of your HIV-positive partner(s) tell you that they were untransmittable or had an undetectable viral load (i.e., U=U)?

( ) Yes

( ) No

( ) Don't know

## Sexual Behavior: Risky Sex

Shortname / Alias: uai_hivunk_p6m

#### ****In the past 6 months****, did you have anal sex WITHOUT a condom with a partner whose HIV status you did not know?

( ) Yes

( ) No

Shortname / Alias: alcdrugsb4sex

#### ****In the past 6 months****, how often did you drink alcohol or use drugs before you had sexual intercourse?

( ) Never

( ) Rarely

( ) Sometimes

( ) Often

( ) Always

## Transition btw Sex Hx & HIV-negative Cascade

### *****On to more serious matters...*****

## HIV-Negative Cascade: PEP

Shortname / Alias: pep_aware

#### Before today, have you ever heard of people taking anti-HIV medicines AFTER a sexual or drug use exposure, to reduce the risk of getting HIV? This is called post-exposure prophylaxis, or PEP.

( ) Yes

( ) No

**Logic: Show/hide trigger exists.**

Shortname / Alias: ever_pep

#### Have you ever taken post-exposure prophylaxis (PEP) AFTER a sexual or drug use exposure, to reduce the risk of getting HIV?

( ) Yes

( ) No

**Logic: Hidden unless: #70 Question "Have you ever taken post-exposure prophylaxis (PEP) AFTER a sexual or drug use exposure, to reduce the risk of getting HIV?" is one of the following answers ("Yes")**

Shortname / Alias: pep_p12m

#### ****In the past 12 months****, have you taken PEP (post-exposure prophylaxis) AFTER a sexual or drug use exposure, to reduce the risk of getting HIV?

( ) Yes

( ) No

## HIV-Negative Cascade: PrEP

Shortname / Alias: prep_aware

#### Before today, have you ever heard of people regularly taking anti-HIV medicines BEFORE a sexual or drug use exposure, to reduce the risk of getting HIV? This is called pre-exposure prophylaxis, or PrEP.

( ) Yes

( ) No

Shortname / Alias: ever_prep

#### Have you ever taken PrEP (pre-exposure prophylaxis) BEFORE a sexual or drug use exposure, to reduce the risk of getting HIV?

( ) Yes, I am on PrEP right now

( ) Yes, I was in the past, but I'm not on PrEP anymore

( ) No, I've never taken PrEP

**Page entry logic:** This page will show when: #73 Question "Have you ever taken PrEP (pre-exposure prophylaxis) BEFORE a sexual or drug use exposure, to reduce the risk of getting HIV?" is one of the following answers ("Yes, I am on PrEP right now","Yes, I was in the past, but I'm not on PrEP anymore")

## HIV-Negative Cascade: PrEP Use

Shortname / Alias: prep_source

#### Did you get your PrEP from the following people or places? (Check all that apply)

[ ] Doctor or other health care provider

[ ] Sex partner, friend, relative, or acquaintance

[ ] Internet

[ ] Other, please specify:: _________________________________________________

**Logic: Hidden by default**

### *****You entered a future date for the month/year you first began taking PrEP. Please correct this.*****

Shortname / Alias: prep_start_yr

#### When did you first begin taking PrEP? Year:

( ) 1992

( ) 1993

( ) 1994

( ) 1995

( ) 1996

( ) 1997

( ) 1998

( ) 1999

( ) 2000

( ) 2001

( ) 2002

( ) 2003

( ) 2004

( ) 2005

( ) 2006

( ) 2007

( ) 2008

( ) 2009

( ) 2010

( ) 2011

( ) 2012

( ) 2013

( ) 2014

( ) 2015

( ) 2016

( ) 2017

( ) 2018

( ) 2019

( ) 2020

( ) 2021

Shortname / Alias: prep_start_mo

#### Month:

( ) January

( ) February

( ) March

( ) April

( ) May

( ) June

( ) July

( ) August

( ) September

( ) October

( ) November

( ) December

**Page entry logic:** This page will show when: #73 Question "Have you ever taken PrEP (pre-exposure prophylaxis) BEFORE a sexual or drug use exposure, to reduce the risk of getting HIV?" is one of the following answers ("No, I've never taken PrEP")

## HIV-Negative Cascade: Barriers to PrEP Use

Shortname / Alias: prepbarrier

#### What are your reasons for not starting PrEP? (Check all that apply)

[ ] I had not heard about PrEP until today

[ ] My work/school schedule could get in the way

[ ] I would forget

[ ] I don't think I need it

[ ] I would have difficulty getting the medication

[ ] I am nervous about side effects that might make me sick

[ ] My friends or family would not support me taking PrEP

[ ] Other, please specify:: _________________________________________________

Shortname / Alias: prep_doc

#### Imagine you were interested in starting PrEP. Do you know of a medical provider that would prescribe PrEP to you?

( ) Yes, definitely

( ) Yes, probably

( ) No, probably not

( ) No, definitely not

## HIV-Negative Cascade: HIV Testing

**Logic: Show/hide trigger exists.**

Shortname / Alias: everhivtest

#### Have you ever had an HIV test?*

( ) Yes

( ) No

( ) Don't know

( ) I prefer not answer

**Logic: Hidden unless: #79 Question "Have you ever had an HIV test?" is one of the following answers ("No")**

Shortname / Alias: reasnotest

#### Which of these best describes the main reason you have not had an HIV test?

( ) I feel at low risk for HIV infection

( ) Afraid of finding out that you had HIV

( ) Haven't had the time

( ) Haven't had the opportunity

( ) Not sure where to get tested

( ) Always know my partner's status, so never needed to get tested

( ) Some other reason, please specify:: _________________________________________________

( ) No particular reason

( ) Don't know

**Logic: Show/hide trigger exists. Hidden unless: #79 Question "Have you ever had an HIV test?" is one of the following answers ("Yes","Don't know")**

Shortname / Alias: hivpos

#### Have you ever tested positive for HIV, that is, do you have HIV? This includes having gotten the virus earlier, but are now suppressed (undetectable).

( ) Yes

( ) No

**Page entry logic:** This page will show when: #79 Question "Have you ever had an HIV test?" is one of the following answers ("Yes","Don't know")

## HIV-Negative Cascade: HIV Testing (cont'd)

**Logic: Hidden by default**

### *****You entered a future date for the month/year of your most recent HIV test. Please correct this.*****

Shortname / Alias: hiv_test_yr

#### When did you have your most recent HIV test? Please tell us the month and year. Year:

( ) 1982

( ) 1983

( ) 1984

( ) 1985

( ) 1986

( ) 1987

( ) 1988

( ) 1989

( ) 1990

( ) 1991

( ) 1992

( ) 1993

( ) 1994

( ) 1995

( ) 1996

( ) 1997

( ) 1998

( ) 1999

( ) 2000

( ) 2001

( ) 2002

( ) 2003

( ) 2004

( ) 2005

( ) 2006

( ) 2007

( ) 2008

( ) 2009

( ) 2010

( ) 2011

( ) 2012

( ) 2013

( ) 2014

( ) 2015

( ) 2016

( ) 2017

( ) 2018

( ) 2019

( ) 2020

Shortname / Alias: hiv_test_mo

#### Month:

( ) January

( ) February

( ) March

( ) April

( ) May

( ) June

( ) July

( ) August

( ) September

( ) October

( ) November

( ) December

Shortname / Alias: hivresult

#### What was the result of your most recent HIV test?

( ) Negative, you do NOT have HIV

( ) Positive, you DO have HIV

( ) Did not receive result

( ) Don't know

**Page entry logic:** This page will show when: (#79 Question "Have you ever had an HIV test?" is one of the following answers ("Yes","Don't know") AND Indicates whether HIV test date is in the future is exactly equal to "0")

## HIV-Negative Cascade: HIV Testing Location

Shortname / Alias: hivloc

#### When you got tested in [question("value"), id="392"]/[question("value"), id="179"], where did you get tested?

( ) HIV counseling and testing site

( ) HIV/AIDS street outreach program or mobile unit

( ) Emergency room

( ) County health clinic or STI program

( ) Private doctor's office (including HMO)

( ) Self tested (collected specimen at home)

( ) Some other place, please specify:: _________________________________________________

**Page entry logic:** This page will show when: #85 Question "When you got tested in [question("value"), id="392"]/[question("value"), id="179"], where did you get tested?" is one of the following answers ("Self tested (collected specimen at home)")

## HIV-Negative Cascade: HIV Self-Testing

### You indicated that you self-tested for HIV. We are interested in learning more about your self-testing experience.

Shortname / Alias: firstselftest

#### Was this the first time you had completed an HIV self-test?

( ) Yes

( ) No

( ) Don't know

Shortname / Alias: selftestloc

#### Where did you get the most recent HIV self-test that you used?

( ) Pharmacy

( ) A sex partner

( ) A friend or family member

( ) Research study or government program

( ) Online

( ) Other, please specify:: _________________________________________________

Shortname / Alias: selftestresults

#### How did you receive your results?

( ) Mailed in specimen and called for results

( ) Read the results myself at home, right after taking the test

Shortname / Alias: selftestwpart

#### When you took your most recent HIV self-test, were you with a sex partner who also took a self-test?

( ) Yes

( ) No

( ) Don't know

**Page entry logic:** This page will show when: #79 Question "Have you ever had an HIV test?" is one of the following answers ("Yes","Don't know")

## HIV-Negative Cascade: HIV Testing Reasons

Shortname / Alias: hivtestreas

#### Have you ever tested for HIV for any of these reasons? (Check all that apply)

[ ] I had unprotected anal sex

[ ] I had unprotected oral sex

[ ] I shared needles or syringes with someone

[ ] I had sex with someone I already knew was HIV positive

[ ] I had sex with someone and found out afterwards that he was HIV positive

[ ] A sex partner requested it

[ ] I was worried I might have been exposed to HIV

[ ] I was sexually assaulted

[ ] I had symptoms of HIV

[ ] I had symptoms of an STI other than HIV

[ ] I get tested on a regular schedule and it was time for me to get tested again

[ ] I was starting a new relationship

[ ] My doctor or other health care provider recommended I get tested

[ ] I received an email, text, or phone call to remind me about testing

[ ] The health department contacted me because one of my sex partners tested positive for HIV

[ ] Because I got paid to have an HIV test as part of a study

[ ] I was somewhere testing was being offered for free (Pride, other testing event)

[ ] Other, please specify:: _________________________________________________

**Page entry logic:** This page will show when: #79 Question "Have you ever had an HIV test?" is one of the following answers ("Yes","Don't know")

## HIV-Negative Cascade: HIV Testing Reasons, Main

Shortname / Alias: mainhivtestreas

#### What is the main reason you got your most recent HIV test?

**Page entry logic:** This page will show when: #79 Question "Have you ever had an HIV test?" is one of the following answers ("Yes","Don't know")

## HIV-Negative Cascade: HIV Testing Habits

Shortname / Alias: reghivtestsched

#### Do you currently test for HIV regularly (after a given amount of time has passed)?

( ) Yes

( ) No

( ) Don't know

Shortname / Alias: hivtestfreq

#### About how often do you test for HIV? Please choose the option that best represents how often you test.

( ) Every month

( ) Every 3 months

( ) Every 6 months

( ) Once a year

( ) Once every 2 years

( ) Other, please specify:: _________________________________________________

Shortname / Alias: hivrecaware

#### As far as you know, what is the current recommendation for HIV testing for men who have sex with other men? Is it recommended they get tested:

( ) Every month

( ) Every 3-6 months

( ) Once a year

( ) Less often than once a year

( ) Don't know

## HIV-Negative Cascade: Serosorting & Other Behaviors

Shortname / Alias: serosort

#### Which of the following describes what you did in the past 12 months to reduce your risk of getting HIV? Check all that apply.

[ ] I did not have sex with HIV-positive men or men whose status I didn't know

[ ] I only had oral sex or jacked off with my partner if he was HIV-positive or I didn't know his status

[ ] I used condoms for anal sex with all of my male partners

[ ] I used condoms for anal sex if my partner was HIV-positive or I didn't know his status

[ ] I topped all my male partners

[ ] I topped my partner if he was HIV-positive or I didn't know his status

[ ] I only had anal sex without a condom with an HIV-positive partner if he was taking HIV medicines and/or he had an undetectable viral load

[ ] I only had anal sex without a condom with an HIV-negative partner if he was taking PrEP

[ ] Other, please specify:: _________________________________________________

[ ] None of these

## HIV Health Literacy

Validation: Min = 0 Max = 100

Shortname / Alias: sexpart_lwh

#### What percent of men would you estimate to be living with HIV among the following groups? Provide your best guess. Sex partners:

0 ________________________[__]_____________________________ 100

Validation: Min = 0 Max = 100

Shortname / Alias: friend_lwh

#### Friends and acquaintances:

0 ________________________[__]_____________________________ 100

Validation: Min = 0 Max = 100

Shortname / Alias: gaybi_lwh

#### Gay and bisexual men where you live:

0 ________________________[__]_____________________________ 100

## HIV Health Literacy, cont'd

Shortname / Alias: hivknow

#### Do you know anyone who has HIV or AIDS?

( ) Yes

( ) No

Shortname / Alias: hivmyth

#### Please read the statements below about HIV. Click the button to indicate if you think the statement is true or false.

|  | **True** | **False** |
| --- | --- | --- |
| A person who has HIV can look healthy. | ( ) | ( ) |
| If a person is infected with HIV, they can show symptoms within a month of being infected. | ( ) | ( ) |
| There is a vaccine that can stop you from getting HIV. | ( ) | ( ) |
| Even if you partner has HIV, the risk for getting HIV is very low when deep kissing (tongue in partner's mouth). | ( ) | ( ) |
| Nearly all HIV transmission comes from having lots of boyfriends or hook-ups. | ( ) | ( ) |
| The risk for getting HIV is very low when having oral sex. | ( ) | ( ) |
| A person is more likely to get HIV from receptive sex (bottom) than insertive sex (top). | ( ) | ( ) |
| Showering or washing your genitals/private parts after having sex will make you less likely to get HIV. | ( ) | ( ) |

## HIV Health Literacy, cont'd

Shortname / Alias: sexhlthinfo

#### Where do you obtain information about your sexual health? (Check all that apply)

[ ] Television

[ ] Newspapers

[ ] Radio

[ ] Online

[ ] Medical journals

[ ] Friends

[ ] Medical provider

[ ] Other, please specify:: _________________________________________________

**Logic: Hidden unless: #81 Question "Have you ever tested positive for HIV, that is, do you have HIV? This includes having gotten the virus earlier, but are now suppressed (undetectable)." is one of the following answers ("No")**

Shortname / Alias: hivprevinfo

#### Where do you obtain information about HIV prevention? (Check all that apply)

[ ] Television

[ ] Newspapers

[ ] Radio

[ ] Online

[ ] Medical journals

[ ] Friends

[ ] Medical provider

[ ] Other, please specify:: _________________________________________________

**Logic: Hidden unless: #81 Question "Have you ever tested positive for HIV, that is, do you have HIV? This includes having gotten the virus earlier, but are now suppressed (undetectable)." is one of the following answers ("No")**

Shortname / Alias: prepinfo

#### Where do you obtain information about PrEP? (Check all that apply)

[ ] Television

[ ] Newspapers

[ ] Radio

[ ] Online

[ ] Medical journals

[ ] Friends

[ ] Medical provider

[ ] Other, please specify:: _________________________________________________

## HIV Health Literacy, cont'd

Shortname / Alias: hivaware

#### Do you feel you have all the information you need about each of the following, or would you like to have more information?

|  | **Have all the information you need** | **Would like to have more information** |
| --- | --- | --- |
| Where to get tested for HIV | ( ) | ( ) |
| How to bring up the topic of getting an HIV test with your partner | ( ) | ( ) |
| How often you should get tested for HIV | ( ) | ( ) |
| How to talk with a health care provider about HIV | ( ) | ( ) |
| How effective condoms are in preventing HIV | ( ) | ( ) |
| Pre-exposure prophylaxis (PrEP), medication taken daily to prevent HIV infection among at risk HIV-negative people | ( ) | ( ) |
| Post-exposure prophylaxis (PEP), medication taken by HIV-negative people within 72 hours of being exposed to HIV to reduce the chance of becoming infected | ( ) | ( ) |
| Treatment as prevention (TasP), antiretroviral treatment (ART) taken by HIV-positive people to decrease the risk of HIV transmission to those that are HIV-negative | ( ) | ( ) |
| When to begin treatment if HIV positive | ( ) | ( ) |
| How risky different sexual behaviors are in terms of HIV transmission | ( ) | ( ) |

**Page entry logic:** This page will show when: (#73 Question "Have you ever taken PrEP (pre-exposure prophylaxis) BEFORE a sexual or drug use exposure, to reduce the risk of getting HIV?" is one of the following answers ("Yes, I am on PrEP right now","Yes, I was in the past, but I'm not on PrEP anymore") OR #72 Question "Before today, have you ever heard of people regularly taking anti-HIV medicines BEFORE a sexual or drug use exposure, to reduce the risk of getting HIV? This is called pre-exposure prophylaxis, or PrEP." is one of the following answers ("Yes"))

## PrEP Effectivness

### PrEP (pre-exposure prophylaxis) is a way to prevent HIV infection. PrEP involves HIV-negative individuals taking anti-HIV medications once a day, every day to reduce the chance of HIV infection if they are exposed to HIV. PrEP is currently available with a prescription and most insurance companies will cover the costs. Please note that PrEP is NOT the same as taking HIV medications for a brief period of time (e.g., 28 days) after a high risk exposure to HIV through situations such as being stuck by a contaminated needle or having unprotected sex. PrEP is intended for regular, long-term use.

Shortname / Alias: prepworks

#### How sure are you the PrEP medication will help protect you from getting HIV infection?

( ) Very sure

( ) Pretty sure

( ) Not sure at all

( ) Don't know

Shortname / Alias: prepeffect

#### Please indicate how much you agree or disagree with the following statements.

|  | **Agree** | **Undecided** | **Disagree** |
| --- | --- | --- | --- |
| PrEP medication does not always protect you from getting HIV infection even if you take it every day. | ( ) | ( ) | ( ) |
| If I take PrEP medication most days but miss a couple of times a week, I am likely to get HIV infection unless I also used condoms every time I have sex. | ( ) | ( ) | ( ) |
| If I take PrEP medication every day, I don't really need to use condoms. | ( ) | ( ) | ( ) |
| It doesn't matter if I miss doses of PrEP medication, as long as I take a pill just before I have sex. | ( ) | ( ) | ( ) |
| If I take PrEP medication every day, I will not get HIV infection no matter how many partners I have even if we don't use a condom. | ( ) | ( ) | ( ) |
| Because I am taking PrEP, I'm not as concerned about condoms slipping and having unsafe sex. | ( ) | ( ) | ( ) |
| Condoms are more effective than PrEP. | ( ) | ( ) | ( ) |
| If I were to take PrEP to prevent HIV, I would be worried about possible medication resistance if I were to become HIV-infected. | ( ) | ( ) | ( ) |

## PrEP Opinions

### ****Please indicate how much you agree or disagree with the following statements.****

Shortname / Alias: prepuseall

#### PrEP should be used by gay, bisexual and all other men who have sex with men to prevent HIV infection.

( ) Strongly agree

( ) Somewhat agree

( ) Neither agree nor disagree

( ) Somewhat disagree

( ) Strongly disagree

Shortname / Alias: prepsafemed

#### PrEP is a safe medication to use to prevent HIV infection.

( ) Strongly agree

( ) Somewhat agree

( ) Neither agree nor disagree

( ) Somewhat disagree

( ) Strongly disagree

Shortname / Alias: prepfund

#### More money should go to research to better understand PrEP as an HIV prevention medication for gay, bisexual and all other men who have sex with men.

( ) Strongly agree

( ) Somewhat agree

( ) Neither agree nor disagree

( ) Somewhat disagree

( ) Strongly disagree

Shortname / Alias: prephiv

#### PrEP should be used by gay, bisexual and all other men who have sex with men who are at a high risk of acquiring HIV to prevent HIV infection.

( ) Strongly agree

( ) Somewhat agree

( ) Neither agree nor disagree

( ) Somewhat disagree

( ) Strongly disagree

## PrEP Opinions (cont'd)

Shortname / Alias: prepben

#### Which of the following do you think would be benefits of taking PrEP? (Check all that apply)

[ ] Taking control of my sexual health

[ ] Increased intimacy with sexual partners

[ ] Increased pleasure during sex

[ ] Less concern about HIV risk

[ ] Less need for condoms

Validation: Min = 0 Max = 100

Shortname / Alias: pctuseprep

#### Among your friends and acquaintances, what proportion are: (Give your best guess.) Using PrEP:

0 ________________________[__]_____________________________ 100

Validation: Min = 0 Max = 100

Shortname / Alias: pctprepsupp

#### Supportive of PrEP use:

0 ________________________[__]_____________________________ 100

Validation: Min = 0 Max = 100

Shortname / Alias: pctprepdiscuss

#### Have discussed PrEP with others:

0 ________________________[__]_____________________________ 100

**Page entry logic:** This page will show when: #73 Question "Have you ever taken PrEP (pre-exposure prophylaxis) BEFORE a sexual or drug use exposure, to reduce the risk of getting HIV?" is one of the following answers ("No, I've never taken PrEP")

## PrEP Attitudes

### ****Please indicate how much you agree or disagree with the following statements.****

Shortname / Alias: prepgay

#### Taking PrEP is a sign of being gay.

( ) Disagree

( ) Somewhat disagree

( ) Neutral

( ) Somewhat agree

( ) Agree

Shortname / Alias: preptop

#### I do not need PrEP if I am a "top."

( ) Disagree

( ) Somewhat disagree

( ) Neutral

( ) Somewhat agree

( ) Agree

Shortname / Alias: prepcomfort

#### I would feel comfortable telling my sexual partners if I used PrEP.

( ) Disagree

( ) Somewhat disagree

( ) Neutral

( ) Somewhat agree

( ) Agree

**Page entry logic:** This page will show when: #73 Question "Have you ever taken PrEP (pre-exposure prophylaxis) BEFORE a sexual or drug use exposure, to reduce the risk of getting HIV?" is one of the following answers ("No, I've never taken PrEP")

## PrEP Attitudes (cont'd)

Shortname / Alias: preptelldoc

#### I would feel comfortable telling my medical providers if I take PrEP.

( ) Disagree

( ) Somewhat disagree

( ) Neutral

( ) Somewhat agree

( ) Agree

Shortname / Alias: preptellfriend

#### I would feel comfortable telling my friends if I take PrEP.

( ) Disagree

( ) Somewhat disagree

( ) Neutral

( ) Somewhat agree

( ) Agree

## PrEP Adherence Self-Efficacy Scale

Validation: Min = 0 Max = 10

Shortname / Alias: prepconfid2

#### We are going to ask you about situations that could occur while taking PrEP. For the following questions we will ask you to tell us how confident you are that you could to do the following things. Use this response scale ranging from 0 ("could not do at all") to 10 ("completely certain could do"). How confident are you that you could:

| Stick to taking PrEP even when side effects begin to interfere with daily activities? | 0 ________________________[__]_____________________________ 10 |
| --- | --- |
| Integrate taking PrEP into your daily routine? | 0 ________________________[__]_____________________________ 10 |
| Integrate taking PrEP into your daily routine even if it means taking medications or doing other things in front of people who don't know you are taking PrEP? | 0 ________________________[__]_____________________________ 10 |
| Stick to your PrEP schedule even when your daily routine is disrupted? | 0 ________________________[__]_____________________________ 10 |
| Stick to your PrEP schedule when you aren't feeling well? | 0 ________________________[__]_____________________________ 10 |
| Continue taking PrEP even if it means doing so interferes with your daily activities? | 0 ________________________[__]_____________________________ 10 |
| Continue taking PrEP even when you are feeling discouraged about your sexual health? | 0 ________________________[__]_____________________________ 10 |
| Continue taking PrEP even when getting to your clinic appointments is a major hassle? | 0 ________________________[__]_____________________________ 10 |
| Continue taking PrEP even when people close to you tell you that they don't think it is doing any good? | 0 ________________________[__]_____________________________ 10 |

**Page entry logic:** This page will show when: #73 Question "Have you ever taken PrEP (pre-exposure prophylaxis) BEFORE a sexual or drug use exposure, to reduce the risk of getting HIV?" is one of the following answers ("No, I've never taken PrEP")

## PrEP Stigma

Shortname / Alias: prepstigma

#### Please indicate how much you agree or disagree with the following statements.

|  | **Strongly agree** | **Agree** | **Neutral** | **Disagree** | **Strongly disagree** |
| --- | --- | --- | --- | --- | --- |
| I would feel ashamed to take PrEP pills in front of others. | ( ) | ( ) | ( ) | ( ) | ( ) |
| Someone taking PrEP should keep their pills hidden. | ( ) | ( ) | ( ) | ( ) | ( ) |
| People experience negative judgment because they take PrEP. | ( ) | ( ) | ( ) | ( ) | ( ) |
| I would have sex with someone who is taking PrEP. | ( ) | ( ) | ( ) | ( ) | ( ) |
| Someone taking PrEP would be seen by others as slutty. | ( ) | ( ) | ( ) | ( ) | ( ) |
| People taking PrEP receive praise for being responsible. | ( ) | ( ) | ( ) | ( ) | ( ) |
| My friends would be supportive of me taking PrEP. | ( ) | ( ) | ( ) | ( ) | ( ) |
| Someone taking PrEP would be treated unfairly by their doctors. | ( ) | ( ) | ( ) | ( ) | ( ) |
| People experience problems when they tell their sex partner(s) they are taking PrEP. | ( ) | ( ) | ( ) | ( ) | ( ) |
| I would feel proud to take PrEP every day. | ( ) | ( ) | ( ) | ( ) | ( ) |
| People taking PrEP experience verbal harassment. | ( ) | ( ) | ( ) | ( ) | ( ) |
| People on PrEP are taking care of their health. | ( ) | ( ) | ( ) | ( ) | ( ) |
| My family would be supportive of me taking PrEP. | ( ) | ( ) | ( ) | ( ) | ( ) |

## HIV Fatalism

Shortname / Alias: fatalism

#### Please indicate how much you agree or disagree with the following statements.

|  | **Strongly agree** | **Agree** | **Disagree** | **Strongly disagree** |
| --- | --- | --- | --- | --- |
| If one is destined to become infected with HIV, there's nothing you can do about it. | ( ) | ( ) | ( ) | ( ) |
| Most of my friends think that getting HIV sooner or later is unavoidable. | ( ) | ( ) | ( ) | ( ) |
| Most of my friends believe they can do something to prevent HIV transmission. | ( ) | ( ) | ( ) | ( ) |
| Concerns about HIV make me anxious about having sex. | ( ) | ( ) | ( ) | ( ) |

**Page entry logic:** This page will show when: #81 Question "Have you ever tested positive for HIV, that is, do you have HIV? This includes having gotten the virus earlier, but are now suppressed (undetectable)." is one of the following answers ("No")

## Perceived HIV Risk

Shortname / Alias: oddshivacq

#### I think my chances of getting infected with HIV are:

( ) Almost zero

( ) Small

( ) Moderate

( ) Large

( ) Very large

Shortname / Alias: hivacqconcern

#### Getting HIV infection is something I am:

( ) Not concerned about

( ) A little concerned about

( ) Moderately concerned about

( ) Concerned about a lot

( ) Extremely concerned about

Shortname / Alias: infxnrate

#### I am concerned about high rates of HIV infection among people like me:

( ) Agree

( ) Undecided

( ) Disagree

**Page entry logic:** This page will show when: #81 Question "Have you ever tested positive for HIV, that is, do you have HIV? This includes having gotten the virus earlier, but are now suppressed (undetectable)." is one of the following answers ("No")

## Perceived HIV Risk (cont'd)

Shortname / Alias: percsev

#### Becoming HIV infected would make my life harder.

( ) Strongly disagree

( ) Disagree

( ) Neutral

( ) Agree

( ) Strongly agree

Shortname / Alias: stinhiv

#### Having a sexually transmitted infection (e.g., gonorrhea, chlamydia, syphilis) can increase my risk for getting HIV.

( ) Strongly disagree

( ) Disagree

( ) Neutral

( ) Agree

( ) Strongly agree

Shortname / Alias: mkchnghiv

#### I would be willing to make a change in my daily life to prevent HIV.

( ) Strongly disagree

( ) Disagree

( ) Neutral

( ) Agree

( ) Strongly agree

## HIV Stigma

### ****Please indicate how much you agree or disagree with the following statements.****

Shortname / Alias: hivprej

#### Most people in my area would discriminate against someone with HIV.

( ) Strongly agree

( ) Agree

( ) Neither agree nor disagree

( ) Disagree

( ) Strongly disagree

( ) Prefer not to answer

( ) Don't know

Shortname / Alias: hivsupp

#### Most people in my area would support the rights of a person with HIV to live and work wherever they wanted to.

( ) Strongly agree

( ) Agree

( ) Neither agree nor disagree

( ) Disagree

( ) Strongly disagree

( ) Prefer not to answer

( ) Don't know

Shortname / Alias: hivnofrnd

#### Most people in my area would not be friends with someone with HIV.

( ) Strongly agree

( ) Agree

( ) Neither agree nor disagree

( ) Disagree

( ) Strongly disagree

( ) Prefer not to answer

( ) Don't know

Shortname / Alias: hivdeserve

#### Most people in my area would think that people who got HIV through sex or drug use have gotten what they deserve.

( ) Strongly agree

( ) Agree

( ) Neither agree nor disagree

( ) Disagree

( ) Strongly disagree

( ) Prefer not to answer

( ) Don't know

## STI Testing & Diagnosis

Shortname / Alias: stitestfreq

#### How often, on average, do you routinely get tested for STIs (not including HIV)?

( ) Every month

( ) Every 3 months

( ) Every 6 months

( ) Every 9 months

( ) Every 12 months

( ) Every 18 months

( ) Every 24 months

( ) Less frequent than every 24 months

( ) I get tested for STIs at some other interval of time

( ) Don't know

( ) Prefer not to answer

**Logic: Show/hide trigger exists.**

Shortname / Alias: bactstidx

#### ****In the past 6 months****, did a doctor, nurse, or other health care worker diagnose you with any of the following? (Check all that apply)

[ ] Syphilis

[ ] Gonorrhea (clap or drip)

[ ] Chlamydia

[ ] None

**Logic: Show/hide trigger exists.**

Shortname / Alias: viralstidx

#### ****In the past 6 months****, did a doctor, nurse, or other health care worker diagnose you for the first time with any of the following?

[ ] Herpes (HSV)

[ ] Genital warts (HPV)

[ ] Any other STI, please specify:: _________________________________________________

[ ] None

**Page entry logic:** This page will show when: (#134 Question "**In the past 6 months**, did a doctor, nurse, or other health care worker diagnose you with any of the following? (Check all that apply)" is not one of the following answers ("None") OR #135 Question "**In the past 6 months**, did a doctor, nurse, or other health care worker diagnose you for the first time with any of the following?" is not one of the following answers ("None"))

## STI Treatment

**Logic: Hidden unless: #134 Question "In the past 6 months, did a doctor, nurse, or other health care worker diagnose you with any of the following? (Check all that apply)" is one of the following answers ("Syphilis")**

Shortname / Alias: syph_meds

#### You said that you were told that you had syphilis. Did you receive medicine to treat your syphilis?

( ) Yes

( ) No

**Logic: Hidden unless: #134 Question "In the past 6 months, did a doctor, nurse, or other health care worker diagnose you with any of the following? (Check all that apply)" is one of the following answers ("Gonorrhea (clap or drip)")**

Shortname / Alias: gonorrhea_meds

#### You said that you were told that you had gonorrhea. Did you receive medicine to treat your gonorrhea?

( ) Yes

( ) No

**Logic: Hidden unless: #134 Question "In the past 6 months, did a doctor, nurse, or other health care worker diagnose you with any of the following? (Check all that apply)" is one of the following answers ("Chlamydia")**

Shortname / Alias: chlamydia_meds

#### You said that you were told that you had chlamydia. Did you receive medicine to treat your chlamydia?

( ) Yes

( ) No

**Logic: Hidden unless: #135 Question "In the past 6 months, did a doctor, nurse, or other health care worker diagnose you for the first time with any of the following?" is one of the following answers ("Herpes (HSV)")**

Shortname / Alias: herpes_meds

#### You said that you were told that you had herpes. Did you receive medicine to treat your herpes?

( ) Yes

( ) No

**Logic: Hidden unless: #135 Question "In the past 6 months, did a doctor, nurse, or other health care worker diagnose you for the first time with any of the following?" is one of the following answers ("Genital warts (HPV)")**

Shortname / Alias: hpv_meds

#### You said that you were told that you had genital warts. Did you receive medicine to treat your genital warts?

( ) Yes

( ) No

**Logic: Hidden unless: #135 Question "In the past 6 months, did a doctor, nurse, or other health care worker diagnose you for the first time with any of the following?" is one of the following answers ("Any other STI, please specify:")**

Shortname / Alias: othviralsti_meds

#### You said that you were told that you had [question("value"), id="300"]. Did you receive medicine to treat your [question("value"), id="300"]?

( ) Yes

( ) No

## Other Health Indicators: Hepatitis C

**Logic: Show/hide trigger exists.**

Shortname / Alias: hepc_screen

#### Have you been screened for hepatitis C?

( ) Yes, tested negative for hepatitis C

( ) Yes, tested positive for hepatitis C

( ) No, never tested

( ) Don't know

**Logic: Show/hide trigger exists. Hidden unless: #142 Question "Have you been screened for hepatitis C?" is one of the following answers ("Yes, tested positive for hepatitis C","No, never tested","Don't know")**

Shortname / Alias: hepc

#### Has a doctor or nurse ever told you that you had hepatitis C?

( ) Yes

( ) No

( ) Don't know

**Logic: Hidden unless: #143 Question "Has a doctor or nurse ever told you that you had hepatitis C?" is one of the following answers ("Yes")**

Shortname / Alias: dthepcdx

#### When were you told that you had hepatitis C?

( ) 6 months ago or less

( ) More than 6 months ago but less than one year ago

( ) At least 1 year but less than 5 years ago

( ) At least 5 years ago but less than 10 years ago

( ) 10 years ago or more

( ) Don't know

## Other Health Indicators: Hepatitis C (cont'd)

**Logic: Show/hide trigger exists. Hidden unless: #143 Question "Has a doctor or nurse ever told you that you had hepatitis C?" is one of the following answers ("Yes")**

Shortname / Alias: hepctx

#### Have you ever taken medicine to treat your hepatitis C infection?

( ) Yes

( ) No

( ) Don't know

**Logic: Hidden unless: #145 Question "Have you ever taken medicine to treat your hepatitis C infection?" is one of the following answers ("Yes")**

Shortname / Alias: hepc_cured

#### Did you doctor tell you that you were cured of your hepatitis C infection after you finished taking your medicine?

( ) Yes

( ) No

( ) Don't know

## Other Health Indicators: Chronic Disease

**Logic: Show/hide trigger exists.**

Shortname / Alias: chronicdz

#### Have you ever been told by a doctor that you have any of the following? (Check all that apply)

[ ] Depression

[ ] Anxiety

[ ] High blood pressure

[ ] Diabetes

[ ] High cholesterol

[ ] None of the above

**Logic: Hidden unless: #147 Question "Have you ever been told by a doctor that you have any of the following? (Check all that apply)" is one of the following answers ("Depression","Anxiety")**

Shortname / Alias: depanx_tx

#### Are you currently being treated for depression or anxiety? (Taking medicine and/or receiving another form of therapy?)

( ) Yes

( ) No

## Condom Attitudes

#### Please rate how much you agree or disagree with the following statements about condoms.

|  | **Strongly disagree** | **Moderately disagree** | **Neutral** | **Moderately agree** | **Strongly agree** |
| --- | --- | --- | --- | --- | --- |
| Condoms are effective at preventing pregnancy | ( ) | ( ) | ( ) | ( ) | ( ) |
| Condoms are effective at preventing sexually transmitted infections | ( ) | ( ) | ( ) | ( ) | ( ) |
| Condoms are effective at preventing HIV | ( ) | ( ) | ( ) | ( ) | ( ) |
| Condoms are comfortable | ( ) | ( ) | ( ) | ( ) | ( ) |
| Condoms are convenient to use | ( ) | ( ) | ( ) | ( ) | ( ) |
| Condoms decrease sexual pleasure | ( ) | ( ) | ( ) | ( ) | ( ) |
| Condoms are easy to obtain | ( ) | ( ) | ( ) | ( ) | ( ) |
| Condoms are something my friends hate | ( ) | ( ) | ( ) | ( ) | ( ) |
| Condoms are something my sexual partners hate | ( ) | ( ) | ( ) | ( ) | ( ) |
| Condoms are exciting | ( ) | ( ) | ( ) | ( ) | ( ) |
| Condoms are embarrassing | ( ) | ( ) | ( ) | ( ) | ( ) |
| Condoms are difficult to discuss with a partner | ( ) | ( ) | ( ) | ( ) | ( ) |
| Condoms are difficult to use | ( ) | ( ) | ( ) | ( ) | ( ) |
| Condoms are messy | ( ) | ( ) | ( ) | ( ) | ( ) |

## Prevention Activities

#### In the past 12 months...

|  | **No** | **Yes** | **Prefer not to answer** | **Don't know** |
| --- | --- | --- | --- | --- |
| Have you gotten any free condoms, not counting those given to you by a friend, relative, or sex partner? | ( ) | ( ) | ( ) | ( ) |
| Have you had a one-on-one conversation with an outreach worker, counselor, or prevention program worker about ways to prevent HIV? Don't count the times where you had a conversation as part of an HIV test. | ( ) | ( ) | ( ) | ( ) |
| Have you been a participant in any organized session(s) involving a small group of people to discuss ways to prevent HIV? Don't include discussions you had with a group of friends. | ( ) | ( ) | ( ) | ( ) |

## Transition btw HIV stuff to Insurance

### *****What a rock star! You're over halfway done!*****

## Health Insurance: Coverage, Coverage History, & Barriers to Coverage

**Logic: Show/hide trigger exists.**

Shortname / Alias: currins

#### Are you currently covered by health insurance? (This includes Medicare or Medicaid.)

( ) Yes

( ) No

( ) Don't know

**Logic: Show/hide trigger exists. Hidden unless: #151 Question "Are you currently covered by health insurance? (This includes Medicare or Medicaid.)" is one of the following answers ("No")**

Shortname / Alias: insp12m

#### Did you have health insurance at any time in the past year?

( ) Yes

( ) No

**Logic: Hidden unless: #151 Question "Are you currently covered by health insurance? (This includes Medicare or Medicaid.)" is one of the following answers ("No")**

Shortname / Alias: barrier_inscov

#### Thinking about why you do not currently have health insurance, do any of these reasons apply to you? (Check all that apply)

[ ] Too busy to sign up

[ ] Cost of medical plan is too high

[ ] Not sure how to sign up

[ ] Do not qualify for a plan

[ ] Might qualify for other programs, such as Medicaid or Medicare

[ ] I am healthy, I don't feel like I need health insurance

[ ] In between coverage or waiting for COBRA to begin

[ ] Parent or guardian does not have insurance or I am not covered on the plan

[ ] Other, please specify:: _________________________________________________

#### The Affordable Care Act ("Obamacare" or ACA) provides a way for many Americans to buy private health insurance. This is done through Healthcare.gov, or the Health Insurance Marketplace. Have you heard of this way to buy health insurance?

( ) Yes

( ) No

**Page entry logic:** This page will show when: #151 Question "Are you currently covered by health insurance? (This includes Medicare or Medicaid.)" is one of the following answers ("Yes","Don't know")

## Health Insurance: Current Insurance

**Logic: Show/hide trigger exists.**

Shortname / Alias: instype

#### What kind of health insurance or coverage do you currently have? (if you are covered by more than one health insurance plan, please tell us about your PRIMARY plan, the once that you mainly use.)

( ) Private health insurance or HMO

( ) Medicaid

( ) Medicare

( ) TRICARE (CHAMPUS)

( ) Veterans Administration Coverage (VA)

( ) Other, please specify:: _________________________________________________

( ) Don't know

**Logic: Hidden unless: #155 Question "What kind of health insurance or coverage do you currently have? (if you are covered by more than one health insurance plan, please tell us about your PRIMARY plan, the once that you mainly use.)" is one of the following answers ("Private health insurance or HMO")**

Shortname / Alias: pvtins_employ

#### Do you get your private health insurance through your employer, a partner's employer, or other family member's employer?

( ) Yes

( ) No

( ) Don't know

**Logic: Hidden unless: (#155 Question "What kind of health insurance or coverage do you currently have? (if you are covered by more than one health insurance plan, please tell us about your PRIMARY plan, the once that you mainly use.)" is one of the following answers ("Private health insurance or HMO") AND #156 Question "Do you get your private health insurance through your employer, a partner's employer, or other family member's employer?" is one of the following answers ("No","Don't know"))**

Shortname / Alias: pvtins_aca

#### Did you purchase this private health insurance through Healthcare.gov, or the Health Insurance Marketplace?

( ) Yes

( ) No

( ) Don't know

## Health Insurance: Coverage Gaps, Change in Insurance, & Medical Care Costs

Validation: Min = 1 Max = 12 Must be numeric Whole numbers only Positive numbers only

**Logic: Hidden unless: #152 Question "Did you have health insurance at any time in the past year?" is one of the following answers ("No")**

Shortname / Alias: insgap

### ****During the past 12 months****, about how many months were you without insurance or health care coverage? If less than one month, enter "1."

_________________________________________________

Shortname / Alias: inschange

#### ****During the past 12 months****, has there been any change in the type of health insurance or coverage that you have?

( ) Yes

( ) No

( ) Don't know

Shortname / Alias: inscost

#### Thinking about your health insurance situation today compared to 1 year ago, is the amount you spend on your medical care less, about the same, or more?

( ) Less

( ) About the same

( ) More

( ) Don't know

## Health Insurance: Medical Bills

Shortname / Alias: medbill

#### ****During the past 12 months****, did you have problems paying or were you unable to pay any medical bills for your own care?

( ) Yes

( ) No

( ) Don't know

Shortname / Alias: medbill_pymtplan

#### Do you currently have any medical bills for your own care that are being paid off over time? (This could include medical bills being paid off with a credit card, through personal loans, or bill paying arrangements with hospitals or other providers. The bills can be from earlier years as well as this year.)

( ) Yes

( ) No

( ) Don't know

## Health Insurance: Access to Care

**Logic: Show/hide trigger exists.**

Shortname / Alias: seendoc_p12m

#### ****In the past 12 months****, have you been to a doctor or nurse for a medical issue (for a check-up, routine care, or specific concern)?

( ) Yes

( ) No

**Logic: Hidden unless: #163 Question "In the past 12 months, have you been to a doctor or nurse for a medical issue (for a check-up, routine care, or specific concern)?" is one of the following answers ("No")**

Shortname / Alias: nomedappt_p12m

#### Is there a reason why you have not been to a doctor or nurse in the past 12 months? (Check all that apply)

[ ] Feel fine / not sick

[ ] Don't know where to go

[ ] Concerned about being able to pay

[ ] Have had bad experiences in the past

[ ] Too busy

[ ] Other, please specify:: _________________________________________________

**Logic: Hidden unless: #163 Question "In the past 12 months, have you been to a doctor or nurse for a medical issue (for a check-up, routine care, or specific concern)?" is one of the following answers ("Yes")**

Shortname / Alias: apptloc

#### Where have you gone to see a doctor or nurse for a medical issue in the past 12 months?

[ ] Primary care physician (doctor)

[ ] Emergency room

[ ] Service organization / Community-based organization

[ ] Student health services

[ ] County health department

[ ] Other physician's (doctor's) office

[ ] Other, please specify:: _________________________________________________

**Page entry logic:** This page will show when: #163 Question "**In the past 12 months**, have you been to a doctor or nurse for a medical issue (for a check-up, routine care, or specific concern)?" is one of the following answers ("Yes")

## Health Insurance: Medical Care: LGBT Sensitization

**Logic: Show/hide trigger exists.**

Shortname / Alias: outdoc

#### Have you told any of the doctors or nurses that cared for you that you have sex with men?

( ) Yes, I told all of them

( ) Yes, I told some of them

( ) No, I have not told any of them

**Logic: Hidden unless: #166 Question "Have you told any of the doctors or nurses that cared for you that you have sex with men?" is one of the following answers ("Yes, I told some of them")**

#### Which of these doctors or nurses have you told?

[ ] None of these

**Logic: Show/hide trigger exists.**

Shortname / Alias: docaskmsm

#### Did any of the doctors or nurses who did not know you had sex with men ask you if you did?

( ) Yes

( ) No

**Logic: Hidden unless: #168 Question "Did any of the doctors or nurses who did not know you had sex with men ask you if you did?" is one of the following answers ("Yes")**

Shortname / Alias: doctellmsm

#### Did you tell all of these doctors or nurses who asked?

( ) Yes, all of them

( ) Yes, some of them

( ) No

**Logic: Hidden unless: (#169 Question "Did you tell all of these doctors or nurses who asked?" is one of the following answers ("No") OR #166 Question "Have you told any of the doctors or nurses that cared for you that you have sex with men?" is one of the following answers ("Yes, I told some of them","No, I have not told any of them"))**

Shortname / Alias: nodoctell

#### Please indicate reasons why you did not tell them:  (Check all that apply)

[ ] I did not think it was not important to tell my health care provider

[ ] I thought the health care provider would make fun of me or treat me differently

[ ] I thought the health care provider would refuse to provide appropriate care for me

[ ] I was uncomfortable talking about having sex with men with my health care provider

[ ] I was uncomfortable talking about sex at all with my health care provider

[ ] I thought friends, family, or other people in the community would find out

[ ] Other, please specify:: _________________________________________________

**Logic: Hidden unless: (#169 Question "Did you tell all of these doctors or nurses who asked?" is one of the following answers ("Yes, all of them") OR #166 Question "Have you told any of the doctors or nurses that cared for you that you have sex with men?" is one of the following answers ("Yes, I told all of them","Yes, I told some of them"))**

Shortname / Alias: docaction

#### After sharing that you have sex with other men, did any doctor or nurse... (Check all that apply)

[ ] Offer counseling on safer practices while having sex with other men

[ ] Avoid you

[ ] Make fun of you or treat you differently

[ ] Refuse to help you

[ ] Offer counseling on how to prevent HIV

[ ] Ask you about anal itching, anal sores

[ ] Offer testing for HIV and STIs

[ ] Suggest you get care at another place with better services for gay or other men who have sex with men

## Health Insurance: Medical Care Avoidance

**Logic: Show/hide trigger exists.**

Shortname / Alias: avoidmed_p6m

#### At any point in the past 12 months, did you want help for a health-related issue but avoided seeking help?

( ) Yes

( ) No

( ) Don't know

**Logic: Hidden unless: #172 Question "At any point in the past 12 months, did you want help for a health-related issue but avoided seeking help?" is one of the following answers ("Yes","Don't know")**

Shortname / Alias: avoidmed_reas

#### For which reasons did you avoid seeking help? (Check all that apply)

[ ] Don't like doctors

[ ] Was scared of learning I had a specific diagnosis or scared of the treatment

[ ] Didn't want my health or testing information or test results to be reported to the government, my employer, or other authorities

[ ] Couldn't afford to get help

[ ] Didn't know where to go

[ ] Some other reason, please specify:: _________________________________________________

## Comfort Discussing Sex, Adherence

Shortname / Alias: comfortdocsex

#### I feel comfortable talking to my medical provider about my sexual behavior and sexual relationships.

( ) Agree

( ) Undecided

( ) Disagree

Shortname / Alias: docjudgesex

#### I worry that my medical provider will make judgments about me for my sexual behavior.

( ) Agree

( ) Undecided

( ) Disagree

## Transition btw Insurance and Substance Use

### This next section will ask questions about your experiences with alcohol and drugs for recreational use beyond what is prescribed or per recommended use instructions. This information is for research purposes only and honesty is appreciated. Your answers will not be shared with any legal authorities. Please be assured that this information with be treated as *****strictly confidential*****.

###

## Substance Use: General Use

**Logic: Show/hide trigger exists.**

Shortname / Alias: subst_p6m

#### ****In the past 6 months****, have you taken any of these substances? (Select all that you have taken in the past 6 months)

[ ] Alcohol (Malt Liquor, Beer, Vodka, etc.)

[ ] Marijuana (Weed, Pot, Grass, Gas, 420, Kush, Purp, Blunt)

[ ] Cocaine (Coke, Snow, Freebase, White Girl, Bump)

[ ] Crack (Rock)

[ ] Ecstasy (Molly, MDMA, E, X, Beans)

[ ] Methamphetamines (Meth, Crystal, Crank, Tina)

[ ] Hallucinogens (PCP, LSD, Acid, Mushrooms)

[ ] Heroin (Smack, Junk, Black Tar, China White)

[ ] GHB (G)

[ ] Opioids or painkillers (Oxy, Percocet, Vicodin)

[ ] Prescription Uppers (Adderall, Ritalin, Dexedrine)

[ ] Prescription Downers (Ativan, Valium, Xanax, Ambien)

[ ] Aspirin (Excedrin)

[ ] Sizzurp (Syrup)

[ ] Synthetic pot (Potpourri, Spice)

[ ] Spray inhalants

[ ] Poppers (Rush, Head Cleaner)

[ ] Ketamine (Special K)

[ ] Speedballs (Heroin and cocaine together)

[ ] Steroids

[ ] Other (not listed), please specify:: _________________________________________________*

[ ] None of the above

## Substance Use: Combining Substances & Drug Names

**Logic: Hidden unless: #176 Question "In the past 6 months, have you taken any of these substances? (Select all that you have taken in the past 6 months)" is not one of the following answers ("None of the above")**

Shortname / Alias: substcombine

#### Of the substances you said you'd taken in the past 6 months, did you ever combine them or take them at the same time?

( ) Yes

( ) No

( ) Don't know

( ) Prefer not to answer

**Page entry logic:** This page will show when: #176 Question "**In the past 6 months**, have you taken any of these substances? (Select all that you have taken in the past 6 months)" is not one of the following answers ("None of the above")

## Substance Use: Frequency

Shortname / Alias: subst_freq

#### Please tell us how often you use [question("piped value")].

( ) Less than once a month

( ) Once a month

( ) More than once a month

( ) Once a week

( ) More than once a week

( ) Once a day

( ) More than once a day

**Page entry logic:** This page will show when: #176 Question "**In the past 6 months**, have you taken any of these substances? (Select all that you have taken in the past 6 months)" is not one of the following answers ("None of the above")

## Substance Use: Age at first use

**Logic: Hidden by default**

### ****You reported an age greater than your current age ([question('value'), id='499'] years). Please correct this.****

Validation: Min = 1 Must be numeric Whole numbers only Positive numbers only

Shortname / Alias: subst_debut_age

### Please tell us how old you were the first time you used [question("piped value")].

_________________________________________________

**Page entry logic:** This page will show when: #176 Question "**In the past 6 months**, have you taken any of these substances? (Select all that you have taken in the past 6 months)" is one of the following answers ("Alcohol (Malt Liquor, Beer, Vodka, etc.)")

## Substance Use: Alcohol

### Please mark the box that best describes your answer to each question.

Shortname / Alias: alc_freq

#### How often do you have a drink containing alcohol?

( ) Never

( ) Monthly or less

( ) 2-4 times a month

( ) 2-3 times a week

( ) 4 or more times a week

Shortname / Alias: numdrinks

#### How many drinks containing alcohol do you have on a typical day when you are drinking?

( ) 1 or 2

( ) 3 or 4

( ) 5 or 6

( ) 7 to 9

( ) 10 or more

**Page entry logic:** This page will show when: #176 Question "**In the past 6 months**, have you taken any of these substances? (Select all that you have taken in the past 6 months)" is one of the following answers ("Alcohol (Malt Liquor, Beer, Vodka, etc.)")

## Substance Use: Alcohol (cont'd)

### Please mark the box that best describes your answer to each question.

Shortname / Alias: alc_risk

####

|  | **Never** | **Less than monthly** | **Monthly** | **Weekly** | **Daily or almost daily** |
| --- | --- | --- | --- | --- | --- |
| How often do you have six or more drinks on one occasion? | ( ) | ( ) | ( ) | ( ) | ( ) |
| How often during the last year have you found that you were not able to stop drinking once you had started? | ( ) | ( ) | ( ) | ( ) | ( ) |
| How often during the last year have you failed to do what was normally expected of you because of drinking? | ( ) | ( ) | ( ) | ( ) | ( ) |
| How often during the last year have you needed a first drink in the morning to get yourself going after a heavy drinking session? | ( ) | ( ) | ( ) | ( ) | ( ) |
| How often during the last year have you had a feeling of guilt or remorse after drinking? | ( ) | ( ) | ( ) | ( ) | ( ) |
| How often during the last year have you been unable to remember what happened the night before because of your drinking? | ( ) | ( ) | ( ) | ( ) | ( ) |

**Page entry logic:** This page will show when: #176 Question "**In the past 6 months**, have you taken any of these substances? (Select all that you have taken in the past 6 months)" is one of the following answers ("Alcohol (Malt Liquor, Beer, Vodka, etc.)")

## Substance Use: Alcohol Reasons

### *Please mark the box that best describes your answer to each question.*

Shortname / Alias: alc_reas

#### How often would you say you drink alcohol for the following reasons?

|  | **Almost never / Never** | **Some of the time** | **Half of the time** | **Most of the time** | **Almost always / Always** |
| --- | --- | --- | --- | --- | --- |
| To forget your worries or problems | ( ) | ( ) | ( ) | ( ) | ( ) |
| Because it helps you when you feel depressed or nervous | ( ) | ( ) | ( ) | ( ) | ( ) |
| Because it's fun | ( ) | ( ) | ( ) | ( ) | ( ) |
| Because you like the feeling | ( ) | ( ) | ( ) | ( ) | ( ) |
| Because you feel more self-confident or sure of yourself | ( ) | ( ) | ( ) | ( ) | ( ) |
| Because it makes social gatherings more fun | ( ) | ( ) | ( ) | ( ) | ( ) |
| Because people want/expect me to | ( ) | ( ) | ( ) | ( ) | ( ) |
| Because there is nothing else to do | ( ) | ( ) | ( ) | ( ) | ( ) |
| To cheer up when you are in a bad mood | ( ) | ( ) | ( ) | ( ) | ( ) |
| To enjoy sex more | ( ) | ( ) | ( ) | ( ) | ( ) |

**Page entry logic:** This page will show when: #176 Question "**In the past 6 months**, have you taken any of these substances? (Select all that you have taken in the past 6 months)" is one of the following answers ("Marijuana (Weed, Pot, Grass, Gas, 420, Kush, Purp, Blunt)")

## Substance Use: Marijuana Reasons

Shortname / Alias: pot_reas

#### How often would you say you use marijuana for the following reasons?

|  | **Almost never / Never** | **Some of the time** | **Half of the time** | **Most of the time** | **Almost always / Always** |
| --- | --- | --- | --- | --- | --- |
| To forget your worries or problems | ( ) | ( ) | ( ) | ( ) | ( ) |
| Because it helps you when you feel depressed or nervous | ( ) | ( ) | ( ) | ( ) | ( ) |
| Because it's fun | ( ) | ( ) | ( ) | ( ) | ( ) |
| Because you like the feeling | ( ) | ( ) | ( ) | ( ) | ( ) |
| Because you feel more self-confident or sure of yourself | ( ) | ( ) | ( ) | ( ) | ( ) |
| Because it makes social gatherings more fun | ( ) | ( ) | ( ) | ( ) | ( ) |
| Because people want/expect me to | ( ) | ( ) | ( ) | ( ) | ( ) |
| Because there is nothing else to do | ( ) | ( ) | ( ) | ( ) | ( ) |
| To cheer up when you are in a bad mood | ( ) | ( ) | ( ) | ( ) | ( ) |
| To enjoy sex more | ( ) | ( ) | ( ) | ( ) | ( ) |

**Page entry logic:** This page will show when: #176 Question "**In the past 6 months**, have you taken any of these substances? (Select all that you have taken in the past 6 months)" is one of the following answers ("Cocaine (Coke, Snow, Freebase, White Girl, Bump)")

## Substance Use: Cocaine Reasons

Shortname / Alias: coke_reas

#### How often would you say you use cocaine for the following reasons?

|  | **Almost never / Never** | **Some of the time** | **Half of the time** | **Most of the time** | **Almost always / Always** |
| --- | --- | --- | --- | --- | --- |
| To forget your worries or problems | ( ) | ( ) | ( ) | ( ) | ( ) |
| Because it helps you when you feel depressed or nervous | ( ) | ( ) | ( ) | ( ) | ( ) |
| Because it's fun | ( ) | ( ) | ( ) | ( ) | ( ) |
| Because you like the feeling | ( ) | ( ) | ( ) | ( ) | ( ) |
| Because you feel more self-confident or sure of yourself | ( ) | ( ) | ( ) | ( ) | ( ) |
| Because it makes social gatherings more fun | ( ) | ( ) | ( ) | ( ) | ( ) |
| Because people want/expect me to | ( ) | ( ) | ( ) | ( ) | ( ) |
| Because there is nothing else to do | ( ) | ( ) | ( ) | ( ) | ( ) |
| To cheer up when you are in a bad mood | ( ) | ( ) | ( ) | ( ) | ( ) |
| To enjoy sex more | ( ) | ( ) | ( ) | ( ) | ( ) |

**Page entry logic:** This page will show when: #176 Question "**In the past 6 months**, have you taken any of these substances? (Select all that you have taken in the past 6 months)" is one of the following answers ("Ecstasy (Molly, MDMA, E, X, Beans)")

## Substance Use: Ecstasy Reasons

Shortname / Alias: ecstasy_reas

#### How often would you say you use ecstasy for the following reasons?

|  | **Almost never / Never** | **Some of the time** | **Half of the time** | **Most of the time** | **Almost always / Always** |
| --- | --- | --- | --- | --- | --- |
| To forget your worries or problems | ( ) | ( ) | ( ) | ( ) | ( ) |
| Because it helps you when you feel depressed or nervous | ( ) | ( ) | ( ) | ( ) | ( ) |
| Because it's fun | ( ) | ( ) | ( ) | ( ) | ( ) |
| Because you like the feeling | ( ) | ( ) | ( ) | ( ) | ( ) |
| Because you feel more self-confident or sure of yourself | ( ) | ( ) | ( ) | ( ) | ( ) |
| Because it makes social gatherings more fun | ( ) | ( ) | ( ) | ( ) | ( ) |
| Because people want/expect me to | ( ) | ( ) | ( ) | ( ) | ( ) |
| Because there is nothing else to do | ( ) | ( ) | ( ) | ( ) | ( ) |
| To cheer up when you are in a bad mood | ( ) | ( ) | ( ) | ( ) | ( ) |
| To enjoy sex more | ( ) | ( ) | ( ) | ( ) | ( ) |

## Substance Use: Injection Drugs

**Logic: Show/hide trigger exists.**

Shortname / Alias: inj_ever

#### Have you ever in your life shot up or injected any drugs other than those prescribed for you? By shooting up, we mean anytime you might have used a needle to inject drugs in your veins, under the skin, or in the muscle.

( ) Yes

( ) No

**Logic: Show/hide trigger exists. Hidden unless: #185 Question "Have you ever in your life shot up or injected any drugs other than those prescribed for you?
By shooting up, we mean anytime you might have used a needle to inject drugs in your veins, under the skin, or in the muscle." is one of the following answers ("Yes")**

Shortname / Alias: inj_freq

#### ****In the past 6 months****, about how often did you inject?

( ) More than once a day

( ) Once a day

( ) More than once a week

( ) Once a week or less

( ) I did not inject in the past 6 months

( ) Don't know

**Logic: Hidden unless: #186 Question "In the past 6 months, about how often did you inject?" is one of the following answers ("More than once a day","Once a day","More than once a week","Once a week or less","Don't know")**

Shortname / Alias: inj_subst

#### ****In the past six months****, when you were injecting, which substances did you inject? (Check all that apply)

[ ] Speedball (heroin and cocaine together)

[ ] Heroin

[ ] Powder cocaine

[ ] Crack cocaine

[ ] Methamphetamine (meth, crystal meth, speed, crank)

[ ] Painkillers (Oxycontin, Dilaudid)

[ ] Other, please specify:: _________________________________________________

**Page entry logic:** This page will show when: #186 Question "**In the past 6 months**, about how often did you inject?" is not one of the following answers ("I did not inject in the past 6 months")

## Substance Use: Needle Sharing (cont'd)

**Logic: Show/hide trigger exists.**

Shortname / Alias: injshare

#### ****In the past 6 months****, have you injected by using needles, syringes, or other drug preparation equipment (works) that had already been used by another person?

( ) Yes

( ) No

**Logic: Hidden unless: #188 Question "In the past 6 months, have you injected by using needles, syringes, or other drug preparation equipment (works) that had already been used by another person?" is one of the following answers ("Yes")**

Shortname / Alias: injpart_hiv

#### Did any of the people that shared their needles with you have a positive HIV status or an HIV status that was unknown to you?

( ) Yes

( ) No

( ) Don't know

## Partner Violence

Shortname / Alias: ipv_p30d

#### ****In the past 30 days****, has a partner harmed you physically, or attempted to harm you physically? This includes hitting you, kicking you, attempting to strangle you, and/or attacking you with a knife, gun or other weapon.

( ) Yes

( ) No

( ) Prefer not to answer

Shortname / Alias: forcedsex_p30d

#### ****In the past 30 days****, has a partner used physical force or threats of force to make you have sex when you did not want to?

( ) Yes

( ) No

( ) Prefer not to answer

Shortname / Alias: emoabuse_p30d

#### ****In the past 30 days****, has a partner harmed you emotionally, or attempted to harm you emotionally? This includes calling you names or putting you down.

( ) Yes

( ) No

( ) Prefer not to answer

## Mental Health: Depression & Anxiety

### Now we're going to ask you some questions about your mood. When answering, please think about how often the following has occurred during the past 2 weeks.

Shortname / Alias: phq4

#### ****Over the past 2 weeks****, how often have you been bothered by any of the following problems?

|  | **Not at all** | **Several Days** | **More than half the days** | **Nearly every day** | **Prefer not to answer** | **Don't know** |
| --- | --- | --- | --- | --- | --- | --- |
| Little interest or pleasure in doing things? | ( ) | ( ) | ( ) | ( ) | ( ) | ( ) |
| Feeling down, depressed, or hopeless? | ( ) | ( ) | ( ) | ( ) | ( ) | ( ) |
| Feeling nervous, anxious, or on edge? | ( ) | ( ) | ( ) | ( ) | ( ) | ( ) |
| Not being able to stop or control worrying? | ( ) | ( ) | ( ) | ( ) | ( ) | ( ) |

## Mental Health: Depression

**Logic: Hidden unless: ((( Question "Little interest or pleasure in doing things?" is not one of the following answers ("Not at all") AND Question "Feeling down, depressed, or hopeless?" is not one of the following answers ("Not at all")) AND Question "Feeling nervous, anxious, or on edge?" is not one of the following answers ("Not at all")) AND Question "Not being able to stop or control worrying?" is not one of the following answers ("Not at all"))**

Shortname / Alias: phq8

#### ****Over the past 2 weeks****, how often have you been bothered by any of the following problems?

|  | **Not at all** | **Several Days** | **More than half the days** | **Nearly every day** | **Prefer not to answer** | **Don't know** |
| --- | --- | --- | --- | --- | --- | --- |
| Trouble falling asleep or staying asleep, or sleeping too much? | ( ) | ( ) | ( ) | ( ) | ( ) | ( ) |
| Feeling tired or having little energy? | ( ) | ( ) | ( ) | ( ) | ( ) | ( ) |
| Poor appetite or overeating? | ( ) | ( ) | ( ) | ( ) | ( ) | ( ) |
| Feeling bad about yourself - or that you are a failure or have let yourself or your family down? | ( ) | ( ) | ( ) | ( ) | ( ) | ( ) |
| Trouble concentrating on things, such as reading the newspaper or watching television? | ( ) | ( ) | ( ) | ( ) | ( ) | ( ) |
| Moving or speaking so slowly that other people could have noticed, or the opposite - being so fidgety or restless that you have been moving around a lot more than usual? | ( ) | ( ) | ( ) | ( ) | ( ) | ( ) |

## Mental Health: Anxiety

**Logic: Hidden unless: ((( Question "Little interest or pleasure in doing things?" is not one of the following answers ("Not at all") AND Question "Feeling down, depressed, or hopeless?" is not one of the following answers ("Not at all")) AND Question "Feeling nervous, anxious, or on edge?" is not one of the following answers ("Not at all")) AND Question "Not being able to stop or control worrying?" is not one of the following answers ("Not at all"))**

Shortname / Alias: gad7

#### ****Over the past 2 weeks****, how often have you been bothered by any of the following problems?

|  | **Not at all** | **Several Days** | **More than half the days** | **Nearly every day** | **Prefer not to answer** | **Don't know** |
| --- | --- | --- | --- | --- | --- | --- |
| Worrying too much about different things? | ( ) | ( ) | ( ) | ( ) | ( ) | ( ) |
| Trouble relaxing? | ( ) | ( ) | ( ) | ( ) | ( ) | ( ) |
| Being so restless that it's hard to sit still? | ( ) | ( ) | ( ) | ( ) | ( ) | ( ) |
| Becoming easily annoyed or irritable? | ( ) | ( ) | ( ) | ( ) | ( ) | ( ) |
| Feeling afraid as if something awful might happen? | ( ) | ( ) | ( ) | ( ) | ( ) | ( ) |

## Mental Health: Resiliency & Self-Efficacy

Shortname / Alias: nida

#### Please read the following questions and check the boxes that indicate how you feel about yourself.

|  | **Not at all true** | **Hardly true** | **Moderately true** | **Exactly true** |
| --- | --- | --- | --- | --- |
| I can always manage to solve difficult problems if I try hard enough. | ( ) | ( ) | ( ) | ( ) |
| If someone opposes me, I can find the means and ways to get what I want. | ( ) | ( ) | ( ) | ( ) |
| It is easy for me to stick to my aims and accomplish my goals. | ( ) | ( ) | ( ) | ( ) |
| I am confident that I could deal efficiently with unexpected events. | ( ) | ( ) | ( ) | ( ) |
| Thanks to my resourcefulness, I know how to handle unforeseen situations. | ( ) | ( ) | ( ) | ( ) |
| I can solve most problems if I invest the necessary effort. | ( ) | ( ) | ( ) | ( ) |
| I can remain calm when facing difficulties because I can rely on my coping abilities. | ( ) | ( ) | ( ) | ( ) |
| When I am confronted with a problem, I can usually find several solutions. | ( ) | ( ) | ( ) | ( ) |
| If I am in trouble, I can usually think of a solution. | ( ) | ( ) | ( ) | ( ) |

## Mental Health: Emotional & Instrumental Support

Shortname / Alias: emosup

#### People sometimes look to others for companionship, assistance, or other types of support. Check the boxes to indicate how often each of the following kinds of support is available to you if you need it.

|  | **Never** | **Rarely** | **Sometimes** | **Usually** | **Always** |
| --- | --- | --- | --- | --- | --- |
| I have someone who will listen to me when I need to talk. | ( ) | ( ) | ( ) | ( ) | ( ) |
| I have someone to confide in or talk to about myself or my problems. | ( ) | ( ) | ( ) | ( ) | ( ) |
| I have someone who makes me feel appreciated. | ( ) | ( ) | ( ) | ( ) | ( ) |
| I have someone to talk with when I have a bad day. | ( ) | ( ) | ( ) | ( ) | ( ) |

Shortname / Alias: infosup

####

|  | **Never** | **Rarely** | **Sometimes** | **Usually** | **Always** |
| --- | --- | --- | --- | --- | --- |
| I have someone to give me good advice about a crisis if I need it. | ( ) | ( ) | ( ) | ( ) | ( ) |
| I have someone to turn to for suggestions about how to deal with a problem. | ( ) | ( ) | ( ) | ( ) | ( ) |
| I have someone to give me information if I need it. | ( ) | ( ) | ( ) | ( ) | ( ) |
| I get useful advice about important things in life. | ( ) | ( ) | ( ) | ( ) | ( ) |

Shortname / Alias: instsup

####

|  | **Never** | **Rarely** | **Sometimes** | **Usually** | **Always** |
| --- | --- | --- | --- | --- | --- |
| Do you have someone to help you if you are confined to bed? | ( ) | ( ) | ( ) | ( ) | ( ) |
| Do you have someone to take you to the doctor if you need it? | ( ) | ( ) | ( ) | ( ) | ( ) |
| Do you have someone to help with your daily chores if you are sick? | ( ) | ( ) | ( ) | ( ) | ( ) |
| Do you have someone to run errands if you need it? | ( ) | ( ) | ( ) | ( ) | ( ) |

## Mental Health: Companionship & Social Isolation

Shortname / Alias: compsup

#### People sometimes look to others for companionship, assistance, or other types of support. Check the boxes to indicate how often each of the following kinds of support is available to you if you need it.

|  | **Never** | **Rarely** | **Sometimes** | **Usually** | **Always** |
| --- | --- | --- | --- | --- | --- |
| Do you have someone with whom to have fun? | ( ) | ( ) | ( ) | ( ) | ( ) |
| Do you have someone with whom to relax? | ( ) | ( ) | ( ) | ( ) | ( ) |
| Do you have someone with whom you can do something enjoyable? | ( ) | ( ) | ( ) | ( ) | ( ) |
| Do you find companionship when you want it? | ( ) | ( ) | ( ) | ( ) | ( ) |

Shortname / Alias: isolat

#### Please check the following boxes to indicate how often the following statements apply to you.

|  | **Never** | **Rarely** | **Sometimes** | **Usually** | **Always** |
| --- | --- | --- | --- | --- | --- |
| I feel left out. | ( ) | ( ) | ( ) | ( ) | ( ) |
| I feel that people barely know me. | ( ) | ( ) | ( ) | ( ) | ( ) |
| I feel isolated from others. | ( ) | ( ) | ( ) | ( ) | ( ) |
| I feel that people are around me but not with me. | ( ) | ( ) | ( ) | ( ) | ( ) |

## Stigma: Outness

**Logic: Show/hide trigger exists.**

Shortname / Alias: out

#### Have you ever told anyone that you are attracted to or have sex with men?

( ) No

( ) Yes

( ) Prefer not to answer

( ) Don't know

**Logic: Hidden unless: #202 Question "Have you ever told anyone that you are attracted to or have sex with men?" is one of the following answers ("Yes","Don't know")**

Shortname / Alias: whoout

#### Which of the following people have you told that you are attracted to or have sex with men?

|  | **No** | **Yes** | **Does not apply** |
| --- | --- | --- | --- |
| Gay, lesbian, or bisexual friends | ( ) | ( ) | ( ) |
| Friends who are not gay, lesbian, or bisexual | ( ) | ( ) | ( ) |
| Family members | ( ) | ( ) | ( ) |
| Health care providers | ( ) | ( ) | ( ) |
| Employer | ( ) | ( ) | ( ) |
| Fellow employees | ( ) | ( ) | ( ) |

Validation: Min = 0 Max = 100

Shortname / Alias: pctfrndlgbt

#### Among your friends and acquaintances, what proportion are LGBT? Provide your best guess.

0 ________________________[__]_____________________________ 100

## Stigma: Stigma & Tolerance

Shortname / Alias: stigma

#### ****During the past 12 months****, have any of the following things happened to you because someone knew or assumes you were attracted to men?*

|  | **No** | **Yes** | **Prefer not to answer** | **Don't know** | **Does not apply** |
| --- | --- | --- | --- | --- | --- |
| You were called names or insulted | ( ) | ( ) | ( ) | ( ) | ( ) |
| You received poorer services than other people in restaurants, stores, other businesses or agencies | ( ) | ( ) | ( ) | ( ) | ( ) |
| You were treated unfairly at work or school | ( ) | ( ) | ( ) | ( ) | ( ) |
| You were denied or given lower quality health care | ( ) | ( ) | ( ) | ( ) | ( ) |
| You were physically attacked or injured | ( ) | ( ) | ( ) | ( ) | ( ) |

Shortname / Alias: tolerant

#### How strongly do you agree or disagree with the following statement: "Most people in my area are tolerant of gays and bisexuals."

( ) Strongly agree

( ) Agree

( ) Neither agree nor disagree

( ) Disagree

( ) Strongly disagree

( ) Prefer not to answer

( ) Don't know

## Social & Online Behavior

Shortname / Alias: perssocfreq

#### ****In the past 12 months****, how often have you gone to a place (not online) where gay men hang out, meet, or socialize? These could include bars, clubs, social organizations, parks, gay businesses, bookstores, sex clubs, etc.

( ) More than once a day

( ) Once a day

( ) More than once a week

( ) Once a week

( ) More than once a month

( ) Once a month

( ) Less than once a month

( ) Never

( ) Prefer not to answer

( ) Don't know

**Logic: Show/hide trigger exists.**

Shortname / Alias: intsoc

#### ****In the past 12 months****, have you used any of the following kinds of internet sites to meet or socialize with gay men? (Check all that apply)

[ ] Social network websites (such as Facebook)

[ ] Dating websites directed towards gay men

[ ] Mobile phone apps (such as gay chat, dating, and hookup apps)

[ ] Prefer not to answer

[ ] Don't know

[ ] None of the above

**Page entry logic:** This page will show when: #208 Question "**In the past 12 months**, have you used any of the following kinds of internet sites to meet or socialize with gay men? (Check all that apply)" is not one of the following answers ("Prefer not to answer","None of the above")

## Online Social Networking

**Logic: Hidden unless: #208 Question "In the past 12 months, have you used any of the following kinds of internet sites to meet or socialize with gay men? (Check all that apply)" is one of the following answers ("Social network websites (such as Facebook)")**

Shortname / Alias: socnetfreq

#### ****In the past 12 months****, how often did you use social network websites (such as Facebook) to meet or socialize with gay men? (Check all that apply)

( ) More than once a day

( ) Once a day

( ) More than once a week

( ) Once a week

( ) More than once a month

( ) Once a month

( ) Less than once a month

( ) Prefer not to answer

( ) Don't know

**Logic: Hidden unless: #208 Question "In the past 12 months, have you used any of the following kinds of internet sites to meet or socialize with gay men? (Check all that apply)" is one of the following answers ("Dating websites directed towards gay men")**

Shortname / Alias: gaydatefreq

#### ****In the past 12 months****, how often have you used dating websites directed towards gay men to meet or socialize with gay men? (Check all that apply)

( ) More than once a day

( ) Once a day

( ) More than once a week

( ) Once a week

( ) More than once a month

( ) Once a month

( ) Less than once a month

( ) Prefer not to answer

( ) Don't know

**Logic: Hidden unless: #208 Question "In the past 12 months, have you used any of the following kinds of internet sites to meet or socialize with gay men? (Check all that apply)" is one of the following answers ("Mobile phone apps (such as gay chat, dating, and hookup apps)")**

Shortname / Alias: appfreq

#### ****In the past 12 months****, how often did you use mobile phone apps (such as gay chat, dating, and hookup apps) to meet or socialize with gay men? (Check all that apply)

( ) More than once a day

( ) Once a day

( ) More than once a week

( ) Once a week

( ) More than once a month

( ) Once a month

( ) Less than once a month

( ) Prefer not to answer

( ) Don't know

## Sex Life Satisfaction

Shortname / Alias: sexsatis

#### How satisfied are you with your current sex life?

( ) Very satisfied

( ) Satisfied

( ) Unsure

( ) Dissatisfied

( ) Very dissatisfied

( ) Don't know

( ) Prefer not to answer

## Thank You!

### Thank you for completing the survey!

###

###

### 
