# Supplementary material for: A Theoretically Based Mobile App to Increase Pre-Exposure Prophylaxis Uptake Among Men Who Have Sex With Men: Protocol for a Randomized Controlled Trial
Source: JMIR Res Protoc. 2020 Feb 21;9(2):e16231. doi: 10.2196/16231 (PMC7060494; doi:10.2196/16231)
Supplement: Multimedia Appendix 2 [file resprot_v9i2e16231_app2.docx]

# Appendix 2. Monthly check-in surveys to measure PrEP eligibility (Intervention arm) and diet and exercise (Control arm).

# HealthMindr: Monthly Check-in (Intervention arm)

## PrEP

**Logic: Show/hide trigger exists.**

Shortname / Alias: startprep

ID: 2

#### Have you started PrEP?

( ) Yes

( ) No

**Page exit logic:** Skipping to PrEP adherence screener**IF:** #1 Question "Have you started PrEP?" is one of the following answers ("Yes") **THEN:** Jump to [page 9 - PrEP Adherence Screener: Currently taking PrEP](#Page16)

## HIV status

**Logic: Show/hide trigger exists.**

Shortname / Alias: lasthivtest

ID: 3

#### When was the last time you were tested for HIV?

( ) Never

( ) More than 6 months ago

( ) Less than 6 months ago

( ) In the past 3 months

**Logic: Hidden unless: #2 Question "When was the last time you were tested for HIV?" is one of the following answers ("More than 6 months ago","Less than 6 months ago","In the past 3 months")**

Shortname / Alias: hivpos

ID: 4

#### What was the result of that test?

( ) Positive

( ) Negative

( ) Don't know

**Page exit logic:** HIV Positive**IF:** #3 Question "What was the result of that test?" is one of the following answers ("Positive") **THEN:** Jump to [page 10 - Thank You!](#Page2) Flag response as complete

**Page entry logic:** This page will show when: #1 Question "Have you started PrEP?" is one of the following answers ("No")

## Sex

**Logic: Show/hide trigger exists.**

Shortname / Alias: aip6m

ID: 5

#### Approximately how many people have you had anal sex with in the past 6 months?

( ) 0

( ) 1

( ) 2 or more

**Logic: Hidden unless: #4 Question "Approximately how many people have you had anal sex with in the past 6 months?" is one of the following answers ("1","2 or more")**

Shortname / Alias: condom

ID: 6

#### About how often do you use condoms when you have anal sex?

( ) Never

( ) Sometimes

( ) Always

**Logic: Show/hide trigger exists. Hidden unless: #4 Question "Approximately how many people have you had anal sex with in the past 6 months?" is one of the following answers ("1","2 or more")**

Shortname / Alias: sexwhiv

ID: 7

#### Are you having sex with someone who you know is living with HIV?

( ) Yes

( ) No

( ) Don't know

**Page entry logic:** This page will show when: #4 Question "Approximately how many people have you had anal sex with in the past 6 months?" is one of the following answers ("1","2 or more")

## Risk

Shortname / Alias: stipos

ID: 8

#### Have you tested positive for any of the following STIs in the past 6 months: chlamydia, gonorrhea, syphilis?

( ) Yes

( ) No

( ) Don't know

Shortname / Alias: exchange

ID: 9

#### Have you ever exchanged sex for money, drugs, or something else of value?

( ) Yes

( ) No

( ) Don't know

Shortname / Alias: pnp

ID: 10

#### In the past 6 months, have you taken drugs such as poppers, methamphetamines, or GHB before or during sex? This is sometimes called chemsex or party and play (PnP).

( ) Yes

( ) No

( ) Don't know

## Recommendation Script

**Action: Custom Script: PrEP Recommendation Algorithm**

**Hidden Value: preprec**

## Recommendations

**Logic: Hidden unless: preprec is exactly equal to "1"**

ID: 14

### Based on your responses, PrEP might be a great option for you. If you're ready to get started then click here to find a PrEP provider in your area.

**Logic: Hidden unless: preprec is exactly equal to "2"**

ID: 15

### Based on your responses, you might be at increased risk for HIV. Talking to a provider is a great way to learn if PrEP is right for you. If you're ready to get started, then click here to find a PrEP provider in your area.

**Logic: Hidden unless: (#4 Question "Approximately how many people have you had anal sex with in the past 6 months?" is one of the following answers ("0") OR preprec is exactly equal to "0")**

ID: 16

### Based on your responses, now may not be the best time to start PrEP. However, if your circumstances change in the future, you may consider PrEP then.

**Logic: Hidden unless: ((((#5 Question "About how often do you use condoms when you have anal sex?" is one of the following answers ("Never","Sometimes") OR #6 Question "Are you having sex with someone who you know is living with HIV?" is one of the following answers ("Yes")) OR #7 Question "Have you tested positive for any of the following STIs in the past 6 months: chlamydia, gonorrhea, syphilis?" is one of the following answers ("Yes","Don't know")) OR #8 Question "Have you ever exchanged sex for money, drugs, or something else of value?" is one of the following answers ("Yes","Don't know")) OR #9 Question "In the past 6 months, have you taken drugs such as poppers, methamphetamines, or GHB before or during sex? This is sometimes called chemsex or party and play (PnP)." is one of the following answers ("Yes","Don't know"))**

ID: 17

### When you talk to your healthcare provider about PrEP you will be tested for HIV and other STIs. If you choose not to talk to a provider, based on your responses, it may still be a good idea to get tested for HIV and other STIs.

**Logic: Hidden unless: (#2 Question "When was the last time you were tested for HIV?" is one of the following answers ("Never") OR #6 Question "Are you having sex with someone who you know is living with HIV?" is one of the following answers ("Don't know"))**

ID: 18

### At this time a good first step is to get an HIV test. To find a location click below.

**Logic: Hidden unless: #6 Question "Are you having sex with someone who you know is living with HIV?" is one of the following answers ("Don't know")**

ID: 19

### You can also order a FREE HIV home testing kit here.

**Page entry logic:** This page will show when: #1 Question "Have you started PrEP?" is one of the following answers ("Yes")

## PrEP Adherence Screener: Currently taking PrEP

Validation: %s format expected

Shortname / Alias: prepstrt_dt

ID: 37

### When did you begin taking PrEP (mm/dd/yyyy)?

_________________________________________________

**Logic: Show/hide trigger exists.**

Shortname / Alias: prpintfreq

ID: 38

#### How often do you intend to take PrEP?

( ) Daily

( ) Only before and after I have sex (sometimes called "event-based dosing")

( ) Some other schedule: _________________________________________________*

Validation: Min = 0 Max = 7 Must be numeric Whole numbers only Positive numbers only

**Logic: Show/hide trigger exists. Hidden unless: #11 Question "How often do you intend to take PrEP?" is one of the following answers ("Daily")**

Shortname / Alias: nmsdprpdaily

ID: 39

### Considering the past 7 days, how many days did you miss taking your PrEP?

_________________________________________________

**Logic: Show/hide trigger exists. Hidden unless: #11 Question "How often do you intend to take PrEP?" is one of the following answers ("Only before and after I have sex (sometimes called "event-based dosing")")**

Shortname / Alias: msdprpevent

ID: 40

#### Considering the past 7 days, were there times that you did not take PrEP before and after having sex?

( ) No

( ) Yes, I missed at least one dose

( ) I have not had sex in the past 7 days

**Logic: Hidden unless: #12 Question "Considering the past 7 days, how many days did you miss taking your PrEP?" is greater than "0"**

Shortname / Alias: whyprepmiss

ID: 41

#### Considering the past 7 days, what are the reasons that you missed taking your medication? (Check all that apply)

[ ] I forgot

[ ] I was too busy and did not have time

[ ] I did not always have the medicine with me (i.e., sleeping away from the home)

[ ] I did not want people to see me taken PrEP

[ ] I had side effects from the medicine

[ ] I was not sexually active

[ ] I took PrEP only when I thought or knew I was going to have sex

[ ] I forgot to take PrEP because I was drunk or high

[ ] I did not pick up my PrEP prescription or refill

[ ] Other, please specify:: _________________________________________________*

**Logic: Hidden unless: #13 Question "Considering the past 7 days, were there times that you did not take PrEP before and after having sex?" is one of the following answers ("Yes, I missed at least one dose")**

Shortname / Alias: whyprepmiss2

ID: 42

#### Considering the past 7 days, what are the reasons that you missed taking your medication? (Check all that apply)

[ ] I forgot

[ ] I was too busy and did not have time

[ ] I did not always have the medicine with me (i.e., sleeping away from the home)

[ ] I did not want people to see me taken PrEP

[ ] I had side effects from the medicine

[ ] I forgot to take PrEP because I was drunk or high

[ ] I did not pick up my PrEP prescription or refill

[ ] Other, please specify: _________________________________________________*

## Thank You!

ID: 1

### Thank you for taking our survey. Your response is very important to us.

# HealthMindr: Monthly Check-in (Control)

## Exercise

Shortname / Alias: weight

ID: 2

#### How satisfied are you with your current weight?

( ) Satisfied

( ) Not satisfied

Shortname / Alias: diet

ID: 3

#### Are you currently on a reduced-calorie or weight loss diet?

( ) Yes

( ) No

Shortname / Alias: exer_days

ID: 4

#### In the past month, how many days per week did you exercise for 30 minutes or more?

( ) 1-2

( ) 3-4

( ) 5-7

Shortname / Alias: activity

ID: 5

#### What type of activity or activities do you typically do for exercise? Select all that apply.

[ ] Lift weights

[ ] Walk

[ ] Jog/Run

[ ] Hike

[ ] Swim

[ ] Dance

[ ] Aerobics

[ ] Pilates

[ ] Team Sports

[ ] Other

## Meds

**Logic: Show/hide trigger exists.**

Shortname / Alias: otcmeds

ID: 6

#### Do you currently take any over the counter medications or supplements?

( ) Yes

( ) No

**Logic: Hidden unless: #5 Question "Do you currently take any over the counter medications or supplements?" is one of the following answers ("Yes")**

Shortname / Alias: otctype

ID: 7

#### What type of over the counter medications or supplements do you take? Select all that apply.

[ ] Vitamins

[ ] Minerals

[ ] Herbs

[ ] Muscle growth supplements

[ ] Other

**Logic: Show/hide trigger exists.**

Shortname / Alias: newmeds

ID: 8

#### Have you started any new medications in the past month that were given to you by a doctor?

( ) Yes

( ) No

**Logic: Hidden unless: #7 Question "Have you started any new medications in the past month that were given to you by a doctor?" is one of the following answers ("Yes")**

Shortname / Alias: newmedname

ID: 9

### Please list the name of the medication or its purpose.

_________________________________________________

## Thank You!

ID: 1

### Thank you for completing this monthly check-in. Your response is very important to us.
